# Supplementary figures and images for: The variability of song variability in zebra finch (Taeniopygia guttata) populations
Source: R Soc Open Sci. 2019 May 15;6(5):190273. doi: 10.1098/rsos.190273 (PMC6549970; doi:10.1098/rsos.190273)

## Wild TGC (Macquarie)

B140

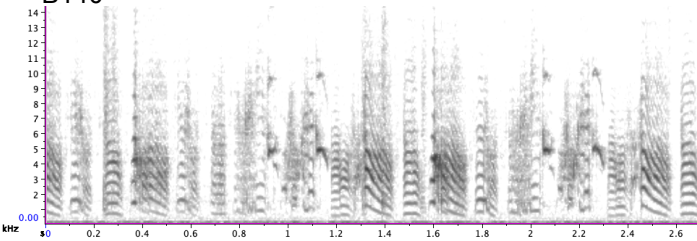

B152

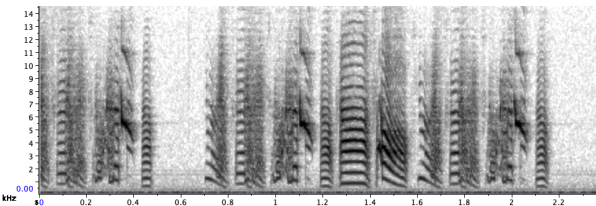

B157

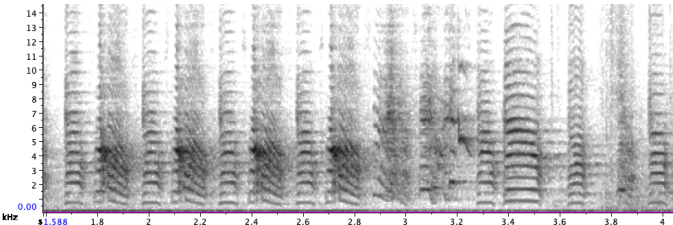

## Domesticated TGC (ECU)

DP99

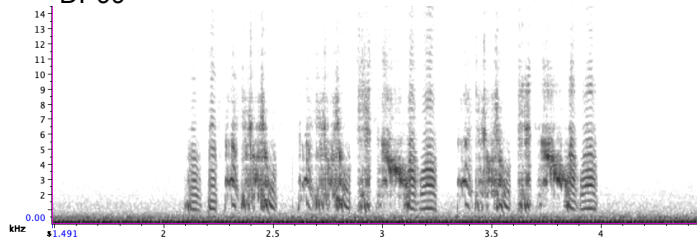

LP47

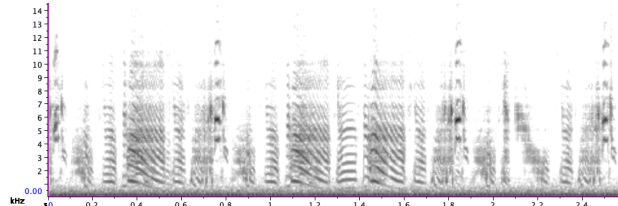

LP49

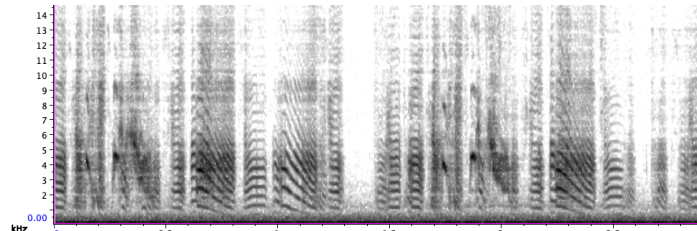

## TGG (ECU)

BL65

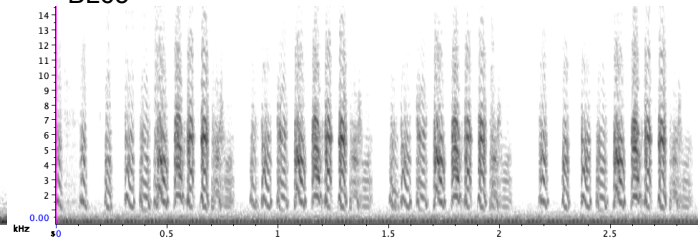

BR82

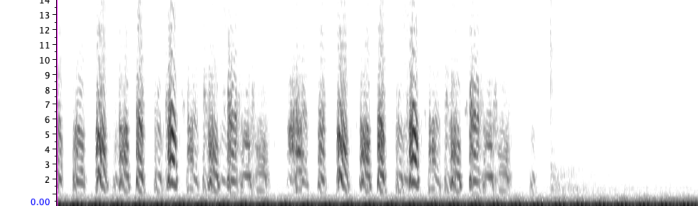

DP51LP79

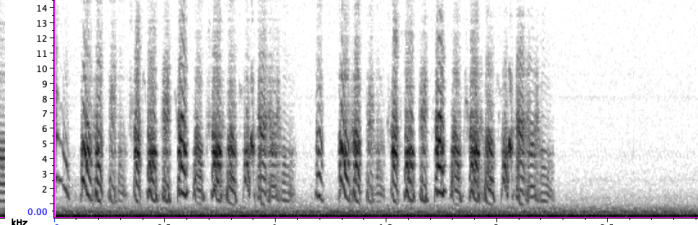

Supplement: Supplemental Figure for The variability of song variability in zebra finch (Taeniopygia guttata) populations [file rsos190273supp1.pdf]
