# Supplementary material for: The variability of song variability in zebra finch (Taeniopygia guttata) populations
Source: R Soc Open Sci. 2019 May 15;6(5):190273. doi: 10.1098/rsos.190273 (PMC6549970; doi:10.1098/rsos.190273)
Supplement: R markdown [file rsos190273supp3.pdf]

Song Variability Analysis

Chris Balakrishnan & Mike McCoy

12/10/2018

R Markdown

This is an R Markdown document. Markdown is a simple formatting syntax for authoring HTML, PDF, and MS Word documents. For more details on using R Markdown see http://rmarkdown.rstudio.com (http://rmarkdown.rstudio.com).

Here we are going to analyze patters of variability is song structure among zebra finch populations. We are going to focus on an analysis of a summary statistic, PC1, derived from the log transformed raw K-L distances. We begin by testing for variation among two domesticated populations of T. guttata castanotis (TGC) [ECU and Chigago], on wild-derived population of TGC (Macquarie University) and one population of T. guttata guttata (TGG; ECU).

```
path<-"~/Documents/Projects/Domestication/OpenBiology/PopulationEffect"
dat=read.table(paste(path,"logKL_4pops_PCA.txt",sep="/"),sep = "\t",header=TRUE)
str(dat)
```

```
## 'data.frame':    278 obs. of  29 variables:
##  $ X                : int  33 34 35 36 37 38 39 40 41 42 ...
##  $ logamplitude     : num  0.0731 0.3528 0.7059 0.3134 0.8614 ...
##  $ logpitch         : num  -0.0401 -0.1553 -0.1624 0.0384 0.0264 ...
##  $ logFM            : num  -0.05112 -0.04725 -0.10572 0.00904 0.20914 ...
##  $ logAM2           : num  -0.0501 0.0308 0.0118 0.0678 0.2659 ...
##  $ logentropy       : num  -0.1463 -0.039 -0.1209 0.0593 -0.0146 ...
##  $ logpitchgoodness : num  0.1776 0.0858 -0.1597 0.1583 0.2722 ...
##  $ logmeanfreq      : num  0.0247 -0.0111 -0.0465 0.1968 0.0784 ...
##  $ logvpitch        : num  -0.284 -0.644 -0.355 -0.212 -0.429 ...
##  $ logvFM           : num  -0.109 -0.305 -0.285 -0.15 -0.19 ...
##  $ logventropy      : num  -0.1256 -0.323 -0.2192 -0.1177 -0.0522 ...
##  $ logvpitchgoodness: num  0.1502 -0.1482 -0.0221 0.1015 0.4843 ...
##  $ vmeanfreq        : num  -0.135 -0.119 -0.17 -0.112 -0.189 ...
##  $ PC1              : num  -4.42 -5.08 -4.86 -3.62 -2.74 ...
##  $ PC1neg           : num  4.42 5.08 4.86 3.62 2.74 ...
##  $ PC2              : num  0.473 -0.739 -1.706 -0.138 -1.931 ...
##  $ PC3              : num  -0.477 -0.42 0.486 -0.172 -0.842 ...
##  $ PC4              : num  -0.1379 -0.227 -0.0764 0.2167 -0.637 ...
##  $ PC5              : num  0.839 0.0795 0.5432 0.5411 0.7339 ...
##  $ PC6              : num  -0.0179 -0.1383 -0.232 0.1277 0.2246 ...
##  $ PC7              : num  -0.4882 0.0517 -0.2967 0.05 -0.5216 ...
##  $ PC8              : num  -0.4904 0.2227 -0.0157 -0.1431 0.1708 ...
##  $ PC9              : num  -0.1904 -0.4548 -0.0159 -0.0331 0.2913 ...
##  $ PC10             : num  -0.346 -0.217 -0.359 -0.372 -0.802 ...
##  $ PC11             : num  0.0963 -0.0212 0.1071 -0.0505 -0.2981 ...
##  $ PC12             : num  0.000142 0.322964 -0.303615 0.046641 -0.440604 ...
##  $ Population       : Factor w/ 4 levels "Chicago","ECU",..: 4 4 4 4 4 4 4 4 4 4 ...
##  $ Template         : Factor w/ 35 levels "filtered_BL65_tclust",..: 1 1 1 1 1 1 1 1 1 2 ...
##  $ Target           : Factor w/ 35 levels "filtered_BL65_tclust",..: 2 3 4 22 23 24 25 26 27 1 ...
```

```
require(ggplot2)
```

```
## Loading required package: ggplot2
```

```
print(ggplot(dat,aes(x=Population,y=PC1))+geom_boxplot())
```

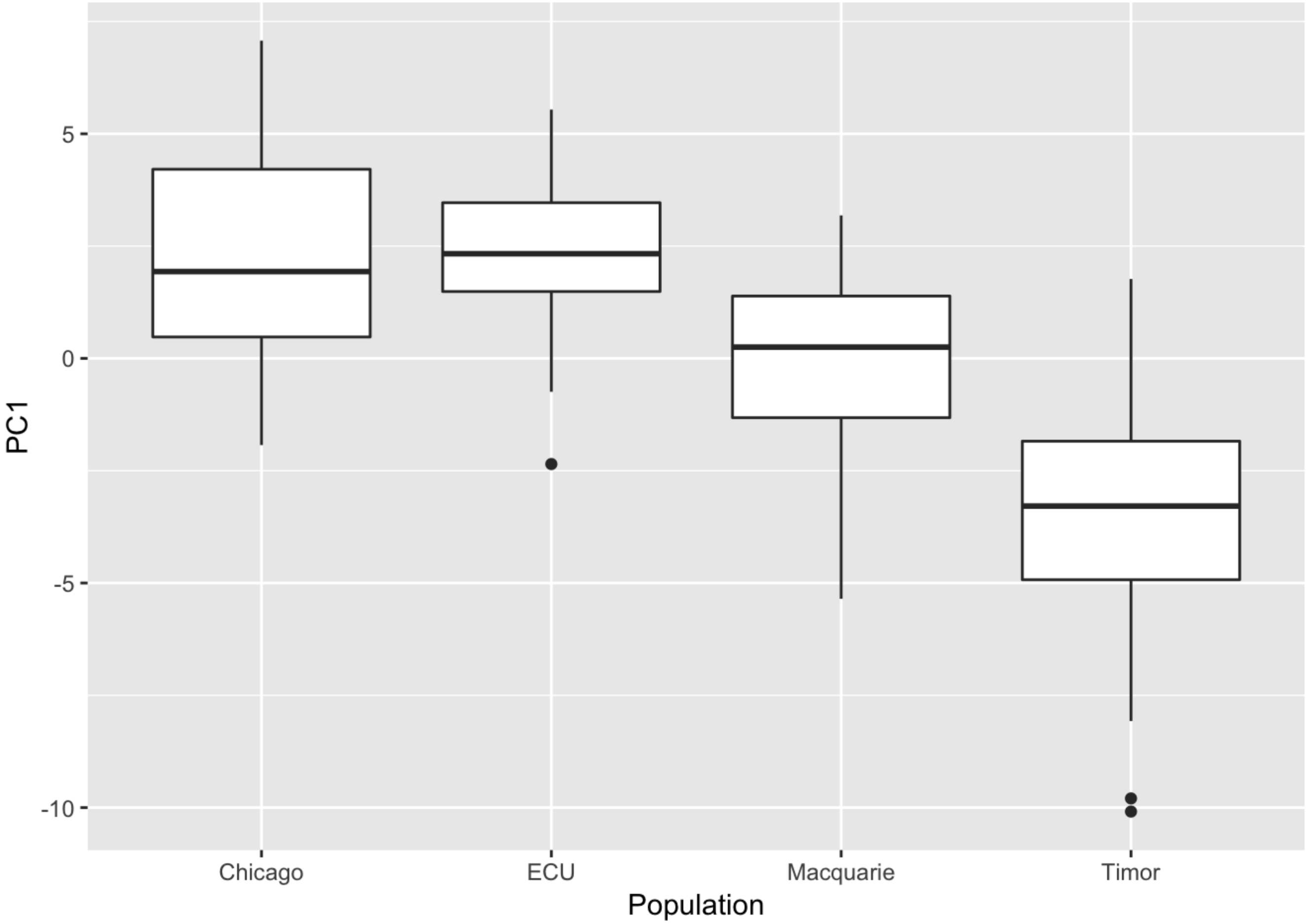

Since KL-distance is a pairwise measurement between two individual birds, we need to deal with the non-independence inherent in the data. TO do this, we will analyze the data using generalized linear mixed models. Each KL measurement includes a “template” and a “target” and we will treat each of these as nested random effects in the model.

We’ve opted to use bayesian mixed models to avoid singularity issues we were having with standard lmer models.

```
require(lme4)
```

```
## Loading required package: lme4
```

```
## Loading required package: Matrix
```

```
library(blme)

mPC1 <- blmer(PC1 ~ Population + (1 |Population/Template) + (1 | Population/Target), data = dat, REML = FALSE)
summary(mPC1)
```

```
## Cov prior  : Target:Population ~ wishart(df = 3.5, scale = Inf, posterior.scale = cov, common.scale = TRUE)
##            : Template:Population ~ wishart(df = 3.5, scale = Inf, posterior.scale = cov, common.scale = TRUE)
##            : Population ~ wishart(df = 3.5, scale = Inf, posterior.scale = cov, common.scale = TRUE)
## Prior dev  : 1.0421
##
## Linear mixed model fit by maximum likelihood  ['blmerMod']
## Formula:
## PC1 ~ Population + (1 | Population/Template) + (1 | Population/Target)
##    Data: dat
##
##      AIC      BIC   logLik deviance df.resid
##   1050.3   1083.0   -516.2   1032.3      269
##
## Scaled residuals:
##     Min      1Q  Median      3Q     Max
## -4.6529 -0.5387  0.1348  0.5755  1.9925
##
## Random effects:
##  Groups              Name        Variance Std.Dev.
##  Target.Population   (Intercept) 1.5569   1.248
##  Template.Population (Intercept) 2.0674   1.438
##  Population          (Intercept) 0.6626   0.814
##  Population.1        (Intercept) 0.6626   0.814
##  Residual                        1.2971   1.139
## Number of obs: 278, groups:
## Target:Population, 35; Template:Population, 35; Population, 4
##
## Fixed effects:
##                     Estimate Std. Error t value
## (Intercept)           2.2516     1.3421   1.678
## PopulationECU         0.1097     1.8717   0.059
## PopulationMacquarie  -2.4293     1.9171  -1.267
## PopulationTimor      -5.5421     1.8717  -2.961
##
## Correlation of Fixed Effects:
##             (Intr) PplECU PpltnM
## PopulatnECU -0.717
## PopultnMcqr -0.700  0.502
## PopulatnTmr -0.717  0.514  0.502
```

```
VarCorr(mPC1) # Check for singularity
```

```
##  Groups              Name        Std.Dev.
##  Target.Population   (Intercept) 1.24777
##  Template.Population (Intercept) 1.43786
##  Population          (Intercept) 0.81398
##  Population.1        (Intercept) 0.81398
##  Residual                        1.13889
```

```
plot(mPC1) # residuals plot
```

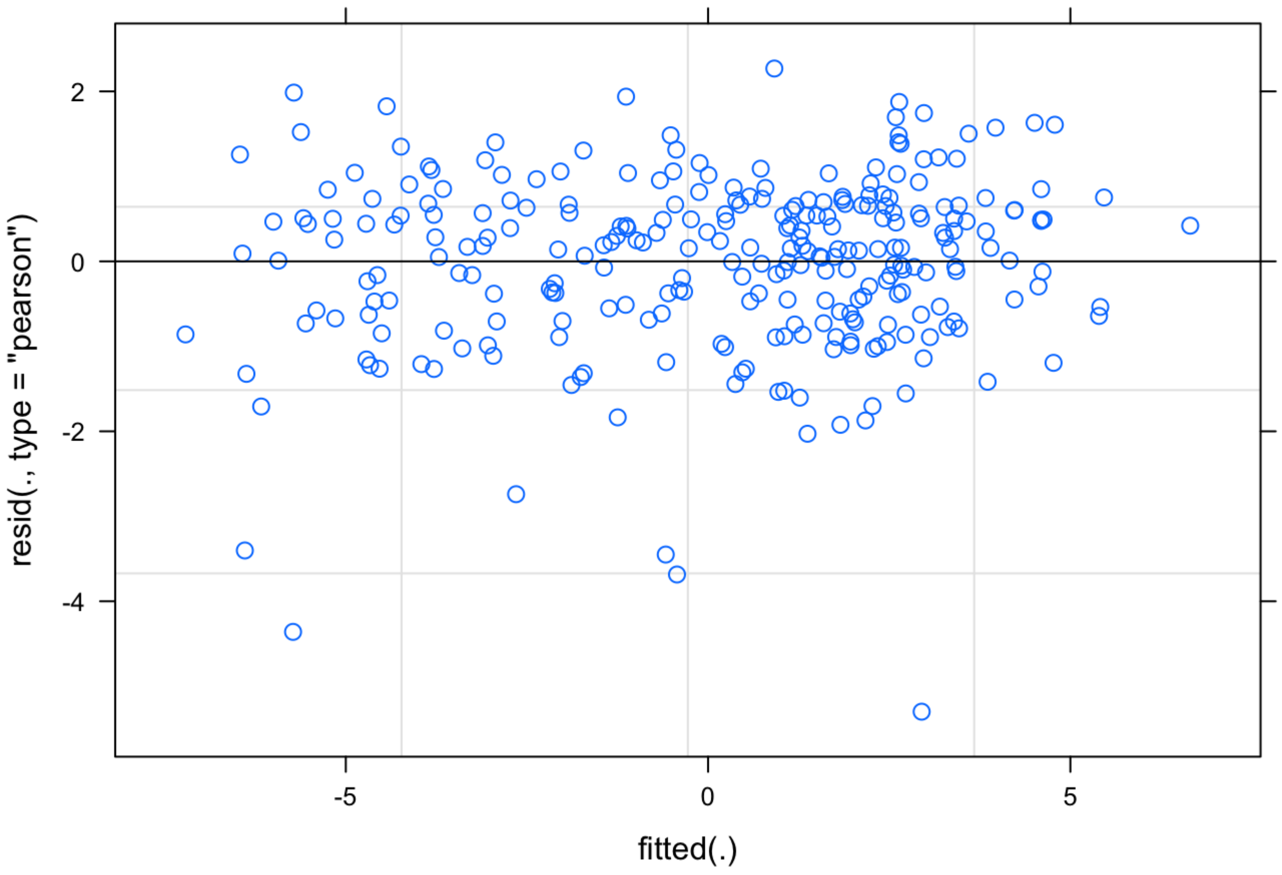

```
qqnorm(resid(mPC1)); qqline(resid(mPC1)) #qqplot
```

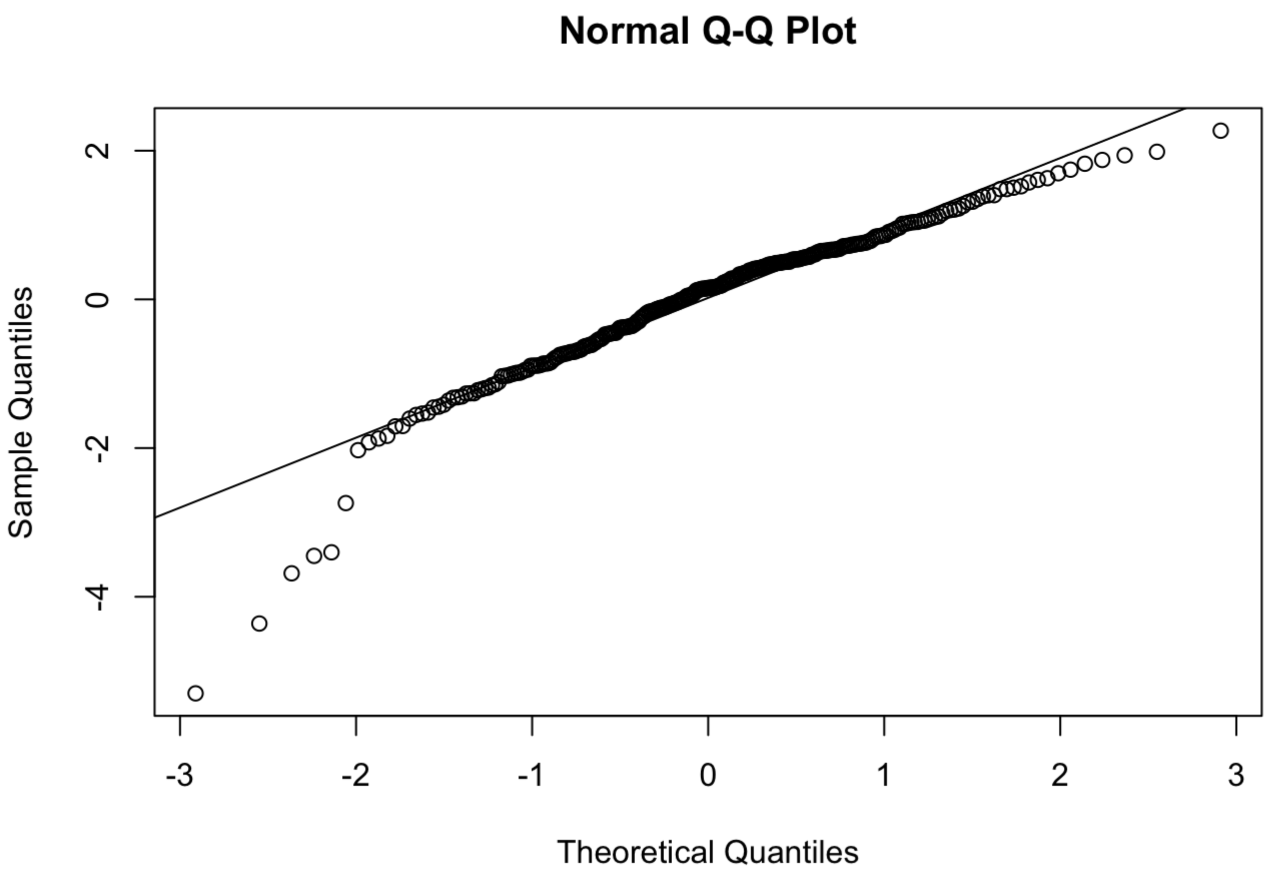


```
#check blmer
```

We can see above that we don’t have an 0 estimates on our error terms. The residuals plot also looks pretty good as does the q-q plot. The lower tail isn’t perfect, but overall this is not too bad.

So now let us fit a null model, removing Population as a fixed factor.

```
mNull <- blmer(PC1 ~ 1 + (1 |Population/Template) + (1 | Population/Target), data = dat, REML = FALSE)
VarCorr(mPC1) # Check for singularity
```

```
##   Groups               Name        Std.Dev.
##   Target.Population    (Intercept) 1.24777
##   Template.Population  (Intercept) 1.43786
##   Population           (Intercept) 0.81398
##   Population.1         (Intercept) 0.81398
##   Residual                         1.13889
```

```
plot(mPC1) # residuals plot
```

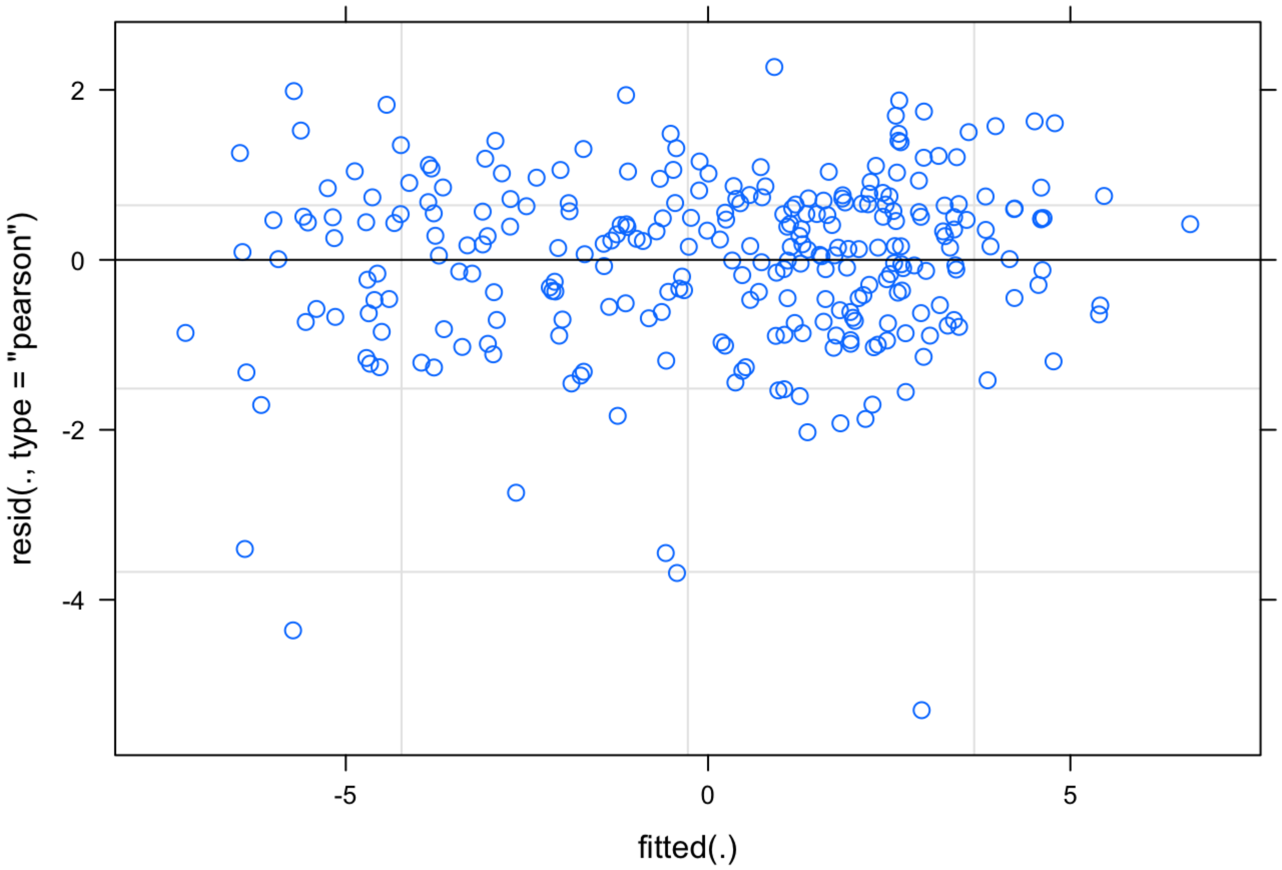


```
qqnorm(resid(mPC1)); qqline(resid(mPC1)) #qqplot
```

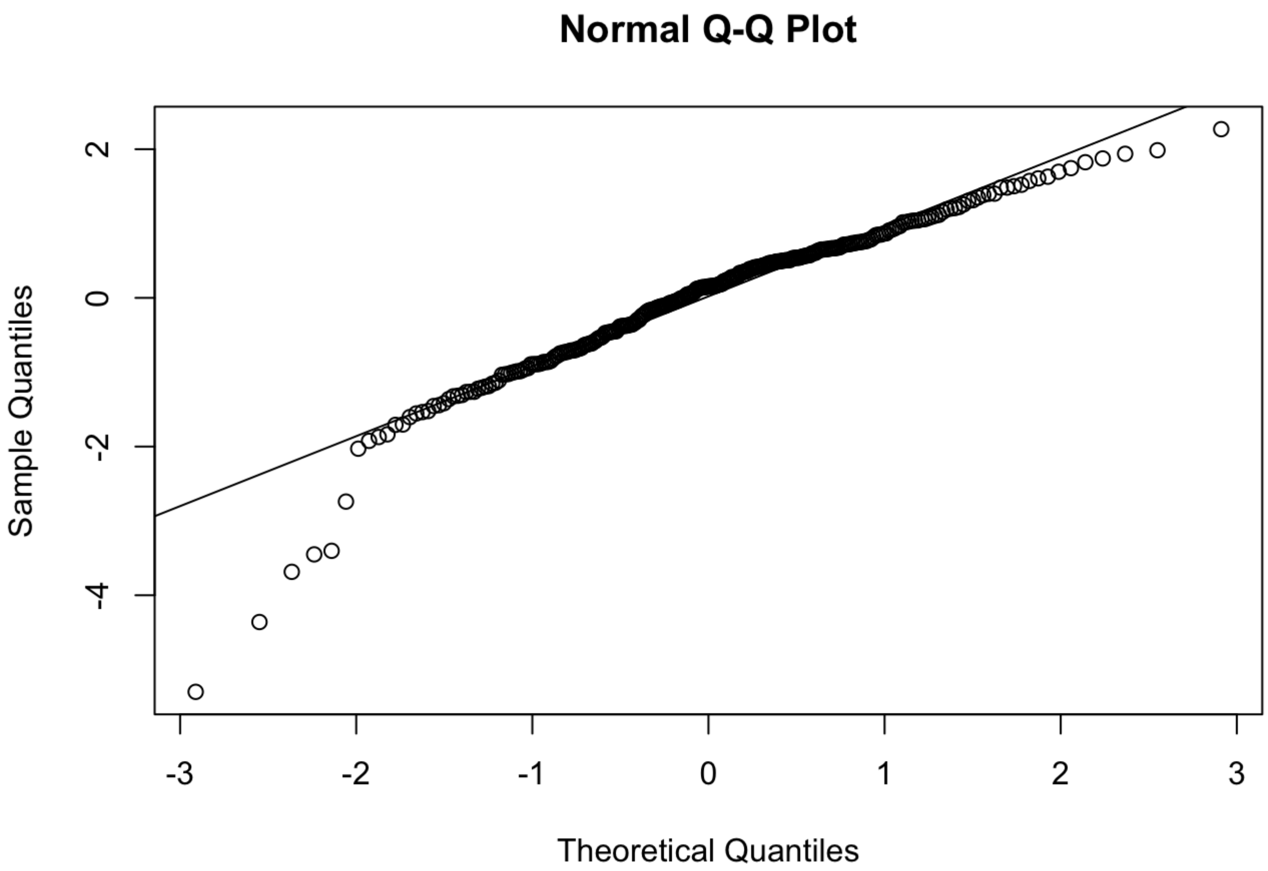

Now lets compare the models using ANOVA

```
anova(mPC1,mNull)
```

```
## Data: dat
## Models:
## mNull: PC1 ~ 1 + (1 | Population/Template) + (1 | Population/Target)
## mPC1: PC1 ~ Population + (1 | Population/Template) + (1 | Population/Target)
##       Df    AIC    BIC  logLik deviance  Chisq Chi Df Pr(>Chisq)
## mNull  6 1055.5 1077.3 -521.76   1043.5
## mPC1   9 1050.3 1083.0 -516.16   1032.3 11.184      3    0.01077 *
## ---
## Signif. codes:  0 '***' 0.001 '**' 0.01 '*' 0.05 '.' 0.1 ' ' 1
```

So there is a significant population effect!!!

Lets load a cheesy little function for extracting the predictions and CIs for plotting.

```
easyPredCInorm <- function(model,newdata,alpha=0.05) {
    ## baseline prediction, on the linear predictor scale:
    pred0 <- predict(model,re.form=NA,newdata=newdata)
    ## fixed-effects model matrix for new data
    X <- model.matrix(formula(model,fixed.only=TRUE)[-2],
                   newdata)
    beta <- fixef(model) ## fixed-effects coefficients
    V <- vcov(model)     ## variance-covariance matrix of beta
    pred.se <- sqrt(diag(X %*% V %*% t(X))) ## std errors of predictions
    ## identity link
    linkinv <- I
    ## construct 95% Normal CIs on the log scale and
    ##  transform back to the response (probability) scale:
    crit <- -qnorm(alpha/2)
    linkinv(cbind(lwr=pred0-crit*pred.se,
                  upr=pred0+crit*pred.se))
}
```

Now we can extract model predicted means and confidence intervals and draw a pretty picture.

```
dat$predicted=(predict(mPC1,re.form=NA))
pframe <- data.frame(Population=dat$Population)
dat$LCI=as.vector((easyPredCInorm(mPC1,pframe))[,1])
dat$UCI=as.vector((easyPredCInorm(mPC1,pframe))[,2])

ggplot(dat,aes(x=Population,y=predicted))+geom_point(size=4)+geom_errorbar(aes(ymin=LCI,ymax=UCI),
                  width=0.4,
                  size=0.5)+
    ylab("PC1 (KL-Distance)")+xlab("Population")+theme_bw() +
  theme(axis.title=element_text(vjust=1,size=16,face="bold"), axis.text=element_text(size=14),
  axis.text.x = element_text(vjust=0.65, hjust=0.5, size=14), panel.border =
  element_rect(colour = "black",size=1.25)) + theme(axis.ticks.length=unit(0.3,"cm"))+
  theme(panel.grid.major = element_blank(), panel.grid.minor =element_blank(), axis.line =
    element_line(colour = "black"))
```

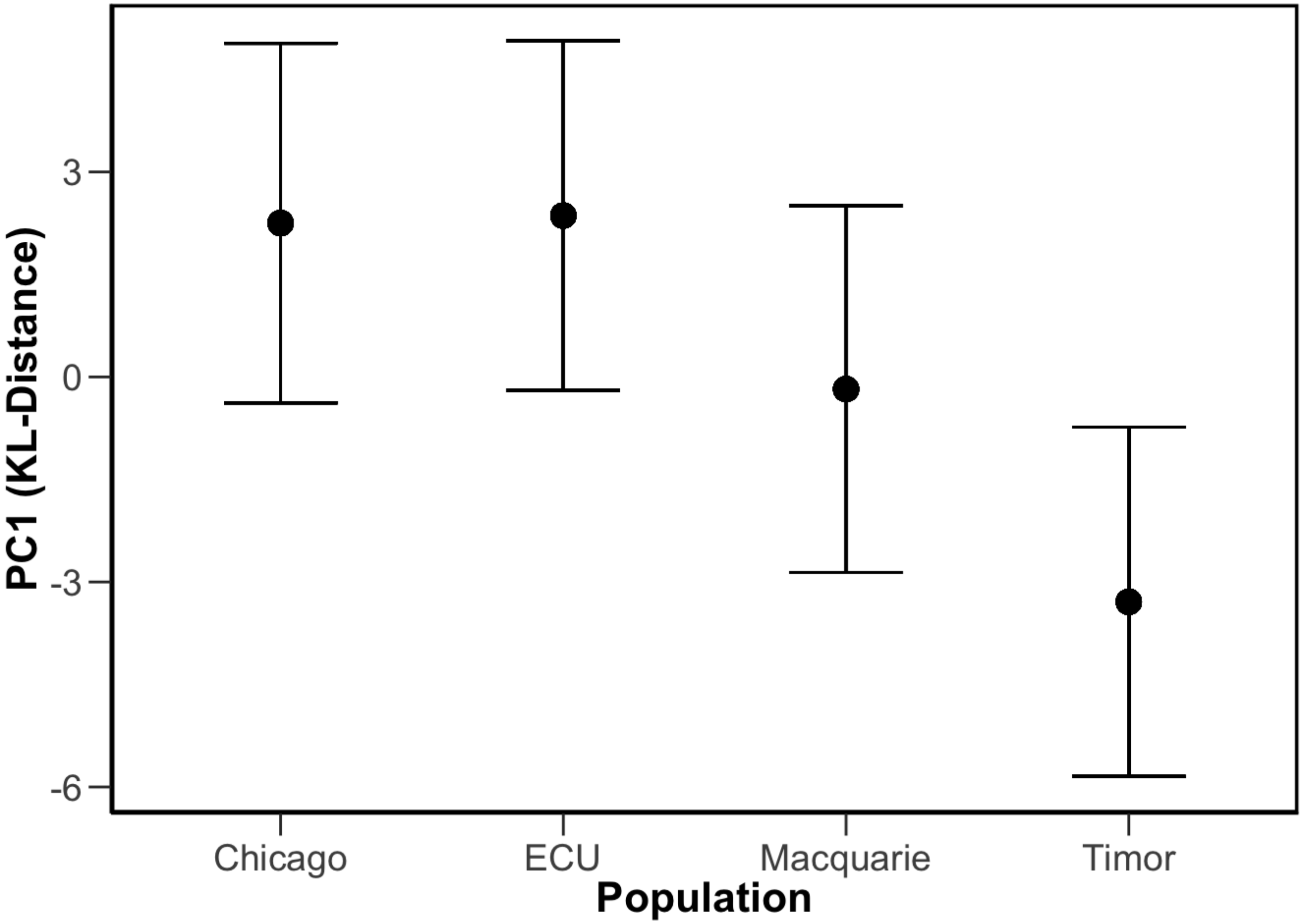


```
#ggsave("PC1-Rplotv_4Pop_Nested.pdf", plot=last_plot(), device=NULL, path=NULL, scale=1, width=NA, height=NA, dpi
=300, limitsize=TRUE)
```

Having demonstrated an overall effect of Population on song variability. We now want to explore this in more detail. As far as we can tell, post hoc tests are not supported for blme, but readers surely will want to know if there is significant variation withing TGC, namely between domesticated an wild. So we drop the Timors from the dataset and run this again.

```
dat2=read.table(paste(path,"logKL_3pops_PCA.txt",sep="/"),sep = "\t",header=TRUE)
str(dat2)
```

```
## 'data.frame':    188 obs. of  30 variables:
##  $ X                : int  123 124 125 126 127 128 129 130 131 132 ...
##  $ logamplitude     : num  1.04 0.943 0.9 1.061 0.951 ...
##  $ logpitch         : num  0.534 0.682 0.766 0.723 0.911 ...
##  $ logFM            : num  0.607 0.719 0.949 0.772 0.826 ...
##  $ logAM2           : num  0.567 0.676 0.773 0.79 0.694 ...
##  $ logentropy       : num  0.542 0.594 0.724 0.746 0.558 ...
##  $ logpitchgoodness : num  0.575 0.694 0.891 0.775 0.939 ...
##  $ logmeanfreq      : num  0.601 0.705 0.723 0.763 0.855 ...
##  $ logvpitch        : num  0.383 0.542 0.547 0.67 0.142 ...
##  $ logvFM           : num  0.511 0.608 0.728 0.756 0.538 ...
##  $ logventropy      : num  0.453 0.61 0.642 0.694 0.54 ...
##  $ logvpitchgoodness: num  0.587 0.632 0.726 0.749 0.772 ...
##  $ vmeanfreq        : num  0.511 0.559 0.614 0.666 0.468 ...
##  $ PC1              : num  2.23 3.2 4.17 4.26 3.55 ...
##  $ PC1neg           : num  -2.23 -3.2 -4.17 -4.26 -3.55 ...
##  $ PC2              : num  -1.147 -0.566 -0.271 -0.684 -0.721 ...
##  $ PC3              : num  0.112 0.0935 -0.4043 0.1929 -1.2095 ...
##  $ PC4              : num  0.0871 0.177 -0.202 0.0909 0.1591 ...
##  $ PC5              : num  0.289 0.184 0.173 0.271 -0.344 ...
##  $ PC6              : num  -0.0182 0.1712 0.2571 -0.019 1.0204 ...
##  $ PC7              : num  -0.0914 -0.2547 -0.1412 -0.1078 -0.1745 ...
##  $ PC8              : num  -0.04286 -0.00937 0.23907 0.09437 -0.13775 ...
##  $ PC9              : num  0.00132 0.15736 0.14971 0.14637 0.25303 ...
##  $ PC10             : num  -0.000771 0.074141 0.377118 -0.054397 -0.124958 ...
##  $ PC11             : num  0.16405 0.05001 -0.03662 0.15893 -0.00725 ...
##  $ PC12             : num  -0.1052 0.016 0.1205 0.0165 0.1192 ...
##  $ Population       : Factor w/ 3 levels "Chicago","ECU",..: 2 2 2 2 2 2 2 2 2 2 ...
##  $ Domesticated     : Factor w/ 2 levels "Domesticated",..: 1 1 1 1 1 1 1 1 1 1 ...
##  $ Template         : Factor w/ 25 levels "filtered_NewRunDP99_tclust",..: 1 1 1 1 1 1 1 1 1 2 ...
##  $ Target           : Factor w/ 25 levels "filtered_NewRunDP99_tclust",..: 2 3 4 5 6 7 8 9 10 1 ...
```

```
print(ggplot(dat2,aes(x=Population,y=PC1))+geom_boxplot())
```

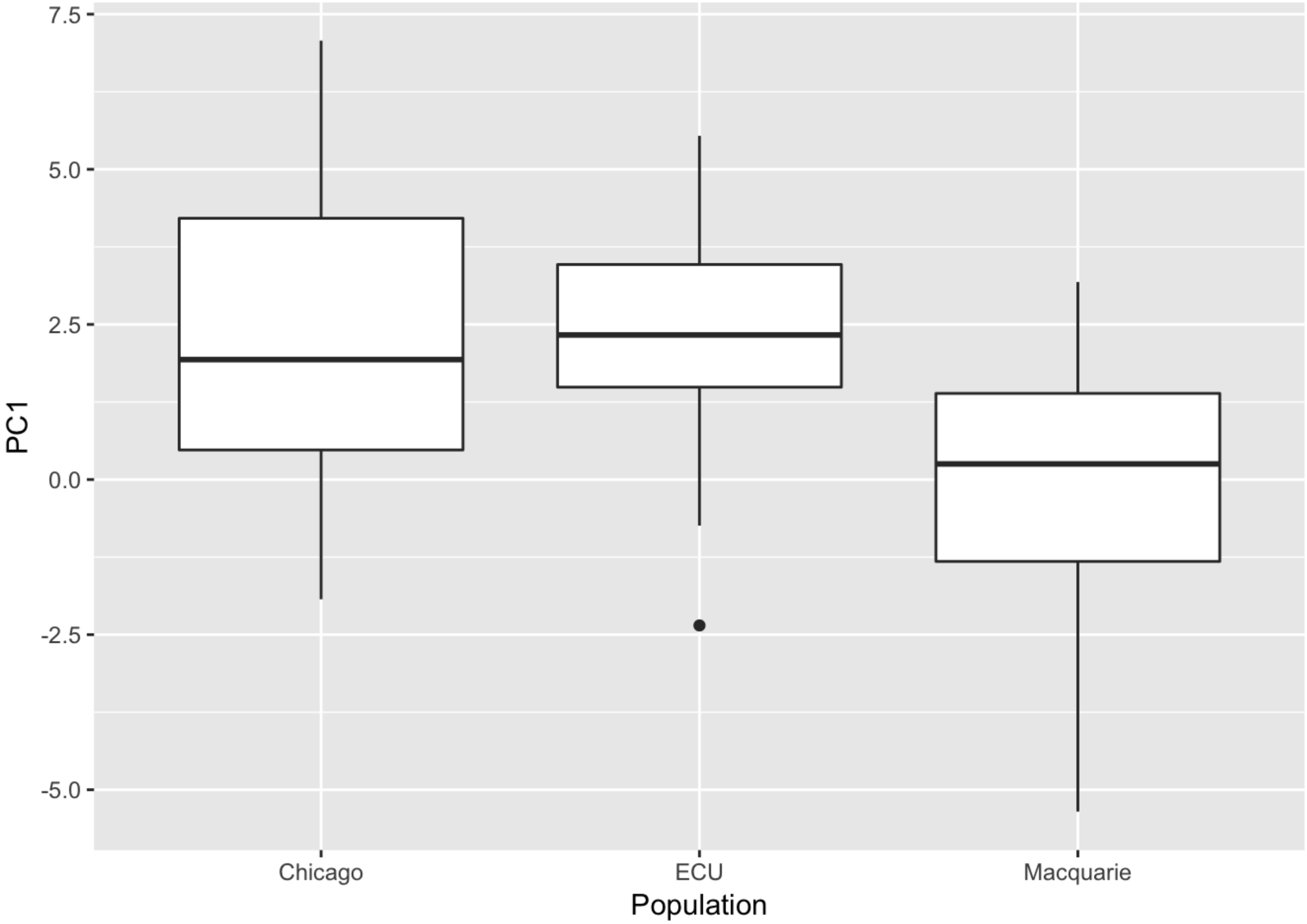

lets build some models

```
mPC1.3pop <- blmer(PC1 ~ Domesticated + (1 |Population/Template) + (1 | Population/Target), data = dat2, REML = F
ALSE)
VarCorr(mPC1.3pop) # Check for singularity
```

```
##  Groups              Name        Std.Dev.
##  Target.Population   (Intercept)  1.4479
##  Template.Population (Intercept)  1.1191
##  Population          (Intercept) 29.6123
##  Population.1        (Intercept) 29.6175
##  Residual                         1.0187
```

```
plot(mPC1.3pop) # residuals plot
```

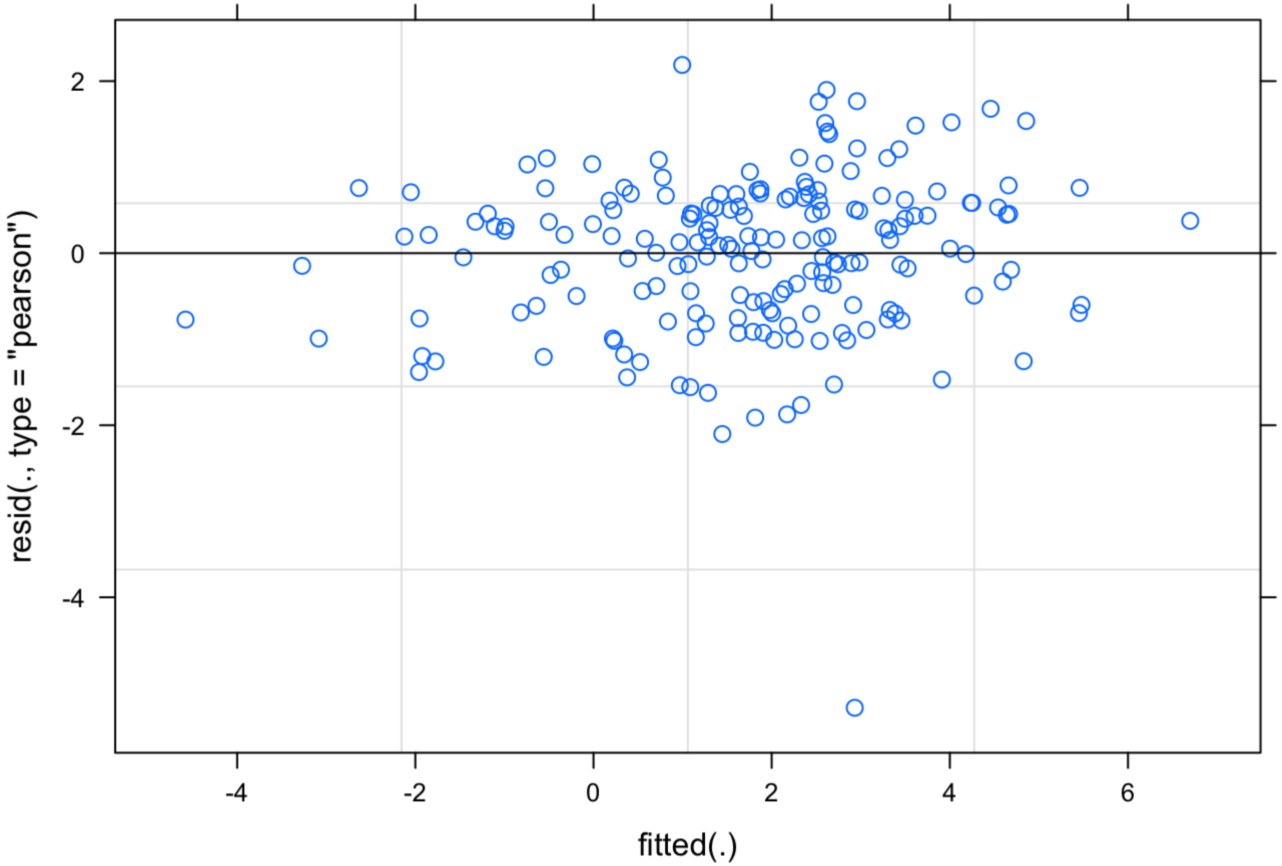


```
qqnorm(resid(mPC1.3pop)); qqline(resid(mPC1.3pop)) #qqplot
```

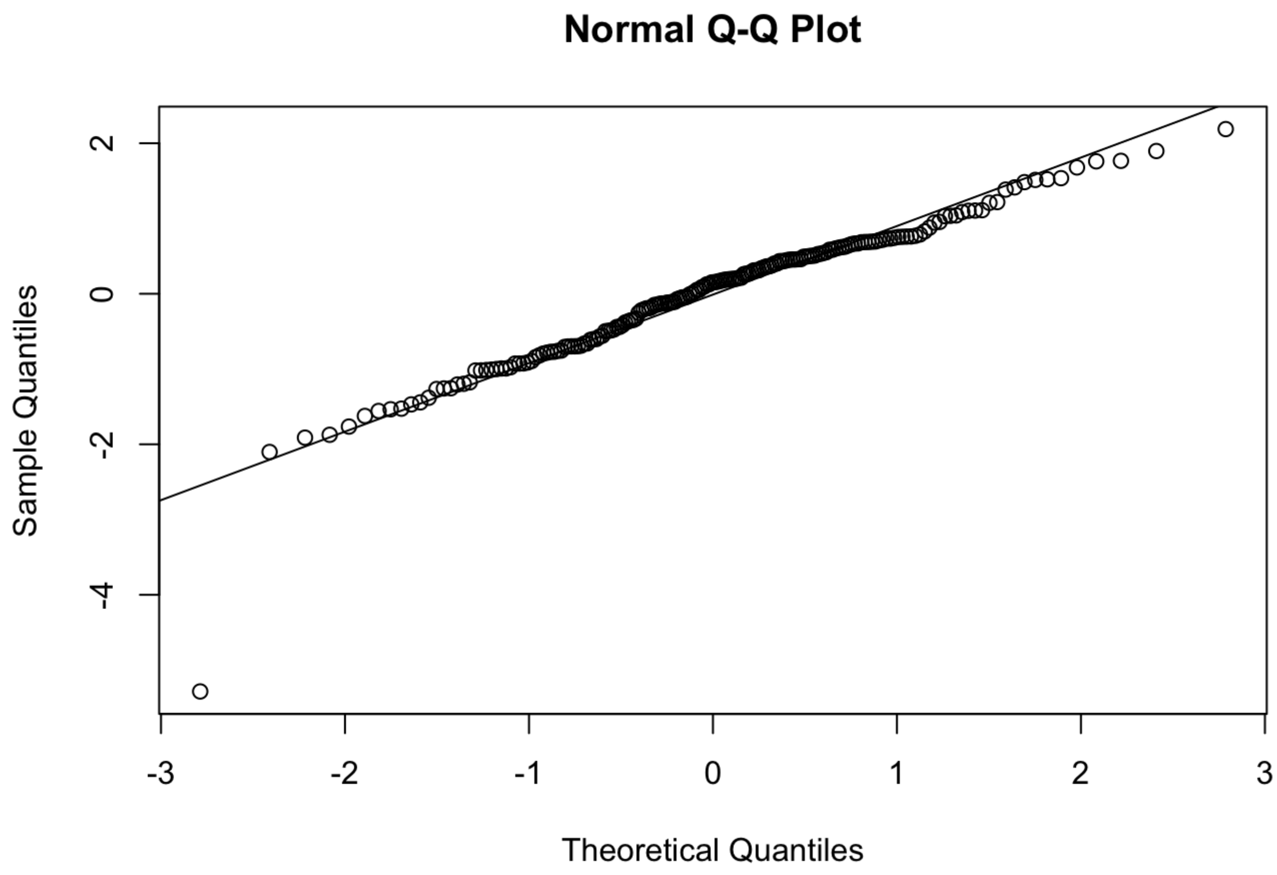


```
mPC1.3popNull <- blmer(PC1 ~ 1 + (1 |Population/Template) + (1 | Population/Target), data = dat2, REML = FALSE)
VarCorr(mPC1.3popNull) # Check for singularity
```

```
##  Groups              Name        Std.Dev.
##  Target.Population   (Intercept)  1.4479
##  Template.Population (Intercept)  1.1191
##  Population          (Intercept) 45.4907
##  Population.1        (Intercept) 45.4931
##  Residual                         1.0187
```

```
plot(mPC1.3popNull) # residuals plot
```

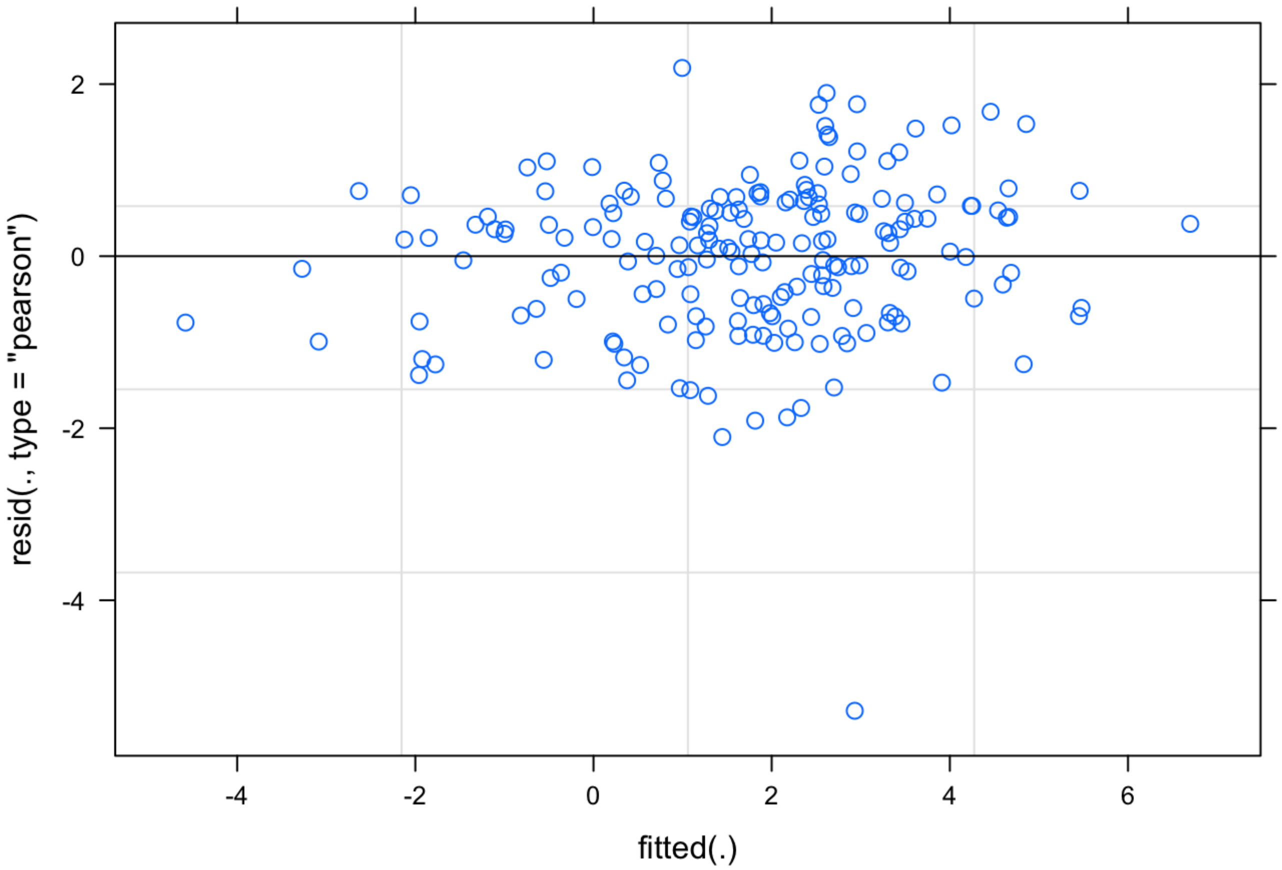


```
qqnorm(resid(mPC1.3popNull)); qqline(resid(mPC1.3popNull)) #qqplot
```

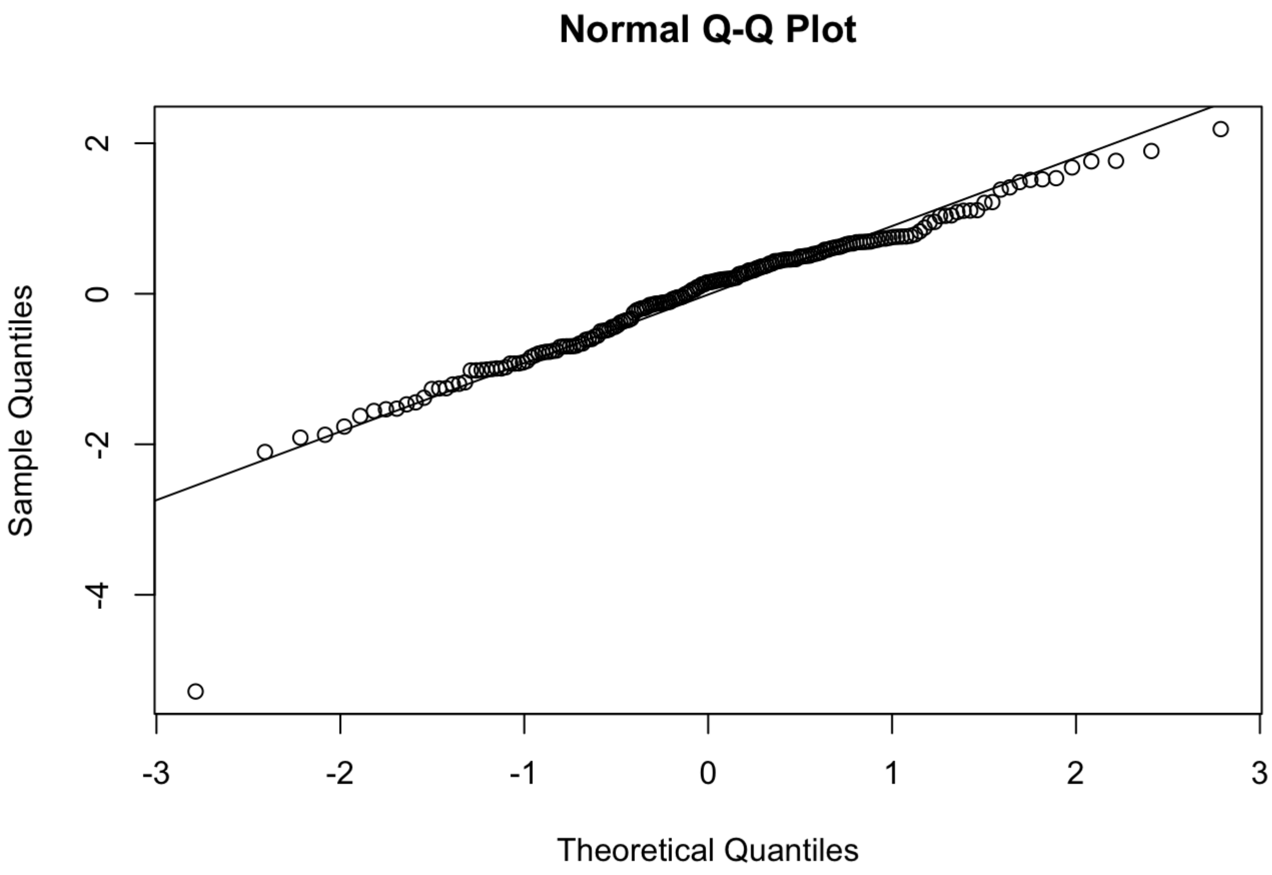

Note that the estimated errors are quite large here, likely do to the small number of levels within the “Population” variabe in the random factors.

```
anova(mPC1.3popNull,mPC1.3pop)
```

```
## Data: dat2
## Models:
## mPC1.3popNull: PC1 ~ 1 + (1 | Population/Template) + (1 | Population/Target)
## mPC1.3pop: PC1 ~ Domesticated + (1 | Population/Template) + (1 | Population/Target)
##               Df    AIC    BIC  logLik deviance  Chisq Chi Df Pr(>Chisq)
## mPC1.3popNull  6 700.96 720.38 -344.48   688.96
## mPC1.3pop      7 700.38 723.04 -343.19   686.38 2.5761      1     0.1085
```

Ok, so a model including “domesticated” as a fixed effect is not better than the null model.

Lastly, we also want to directly compare the two subspecies. This is not strictly a post-hoc test in this case. Here we are able to incorporate additional data. For each of the two subspecies, TGG and TGC we have a small sample of additional data from the UIUC. This data allows to determine whether the low variability observed above has been stable over time/across populations.

```
path<-"~/Documents/Projects/Domestication/OpenBiology/Subspecies"
dat3=read.table(paste(path,"logKL_TvA_PCA.txt",sep="/"),sep = "\t",header=TRUE)
str(dat3)
```

```
## 'data.frame':    212 obs. of  31 variables:
##  $ X                : int  1 2 3 4 5 6 7 8 9 10 ...
##  $ logamplitude     : num  0.593 0.652 0.478 0.294 0.478 ...
##  $ logpitch         : num  0.713 0.721 0.497 0.179 0.421 ...
##  $ logFM            : num  0.611 0.725 0.448 0.23 0.373 ...
##  $ logAM2           : num  0.625 0.719 0.615 0.398 0.447 ...
##  $ logentropy       : num  0.617 0.724 0.456 0.247 0.361 ...
##  $ logpitchgoodness : num  0.721 0.665 0.594 0.33 0.585 ...
##  $ logmeanfreq      : num  0.621 0.691 0.447 0.407 0.491 ...
##  $ logvpitch        : num  0.5507 0.6487 0.4843 0.0129 0.3282 ...
##  $ logvFM           : num  0.5171 0.6269 0.4334 0.0189 0.4684 ...
##  $ logventropy      : num  0.492 0.676 0.5417 -0.0239 0.4716 ...
##  $ logvpitchgoodness: num  0.607 0.552 0.463 0.171 0.521 ...
##  $ vmeanfreq        : num  0.471 0.546 0.408 -0.174 0.306 ...
##  $ PC1              : num  2.489 3.208 1.314 -2.006 0.758 ...
##  $ PC1neg           : num  -2.489 -3.208 -1.314 2.006 -0.758 ...
##  $ PC2              : num  0.448 0.479 0.612 0.309 0.454 ...
##  $ PC3              : num  0.0136 0.368 0.3479 -0.3448 -0.1312 ...
##  $ PC4              : num  0.029 0.1011 -0.1367 0.4306 0.0731 ...
##  $ PC5              : num  0.382 0.0571 0.3869 0.5023 0.5184 ...
##  $ PC6              : num  0.5253 0.1652 0.0143 0.3325 -0.1171 ...
##  $ PC7              : num  -0.017 -0.0171 -0.4781 0.1454 -0.4093 ...
##  $ PC8              : num  0.0176 0.1835 0.0938 0.6944 -0.3397 ...
##  $ PC9              : num  0.0264 0.2188 0.02 0.5259 0.1631 ...
##  $ PC10             : num  -0.0851 0.1441 -0.2972 -0.3736 -0.3687 ...
##  $ PC11             : num  0.0508 -0.067 -0.0722 -0.1573 0.129 ...
##  $ PC12             : num  0.1568 0.0736 0.2731 0.2577 0.1433 ...
##  $ batch            : Factor w/ 2 levels "ECU","UIUC": 2 2 2 2 2 2 2 2 2 2 ...
##  $ Population       : Factor w/ 2 levels "TGC","TGG": 1 1 1 1 1 1 1 1 1 1 ...
##  $ batchpop         : Factor w/ 4 levels "ECU-TGC","ECU-TGG",..: 3 3 3 3 3 3 3 3 3 3 ...
##  $ Template         : Factor w/ 29 levels "filtered_BL65_tclust",..: 15 15 15 16 16 16 17 17 17 18 ...
##  $ Target           : Factor w/ 29 levels "filtered_BL65_tclust",..: 16 17 18 15 17 18 15 16 18 15 ...
```

```r
require(ggplot2)
print(ggplot(dat3,aes(x=batchpop,y=PC1))+geom_boxplot())
```

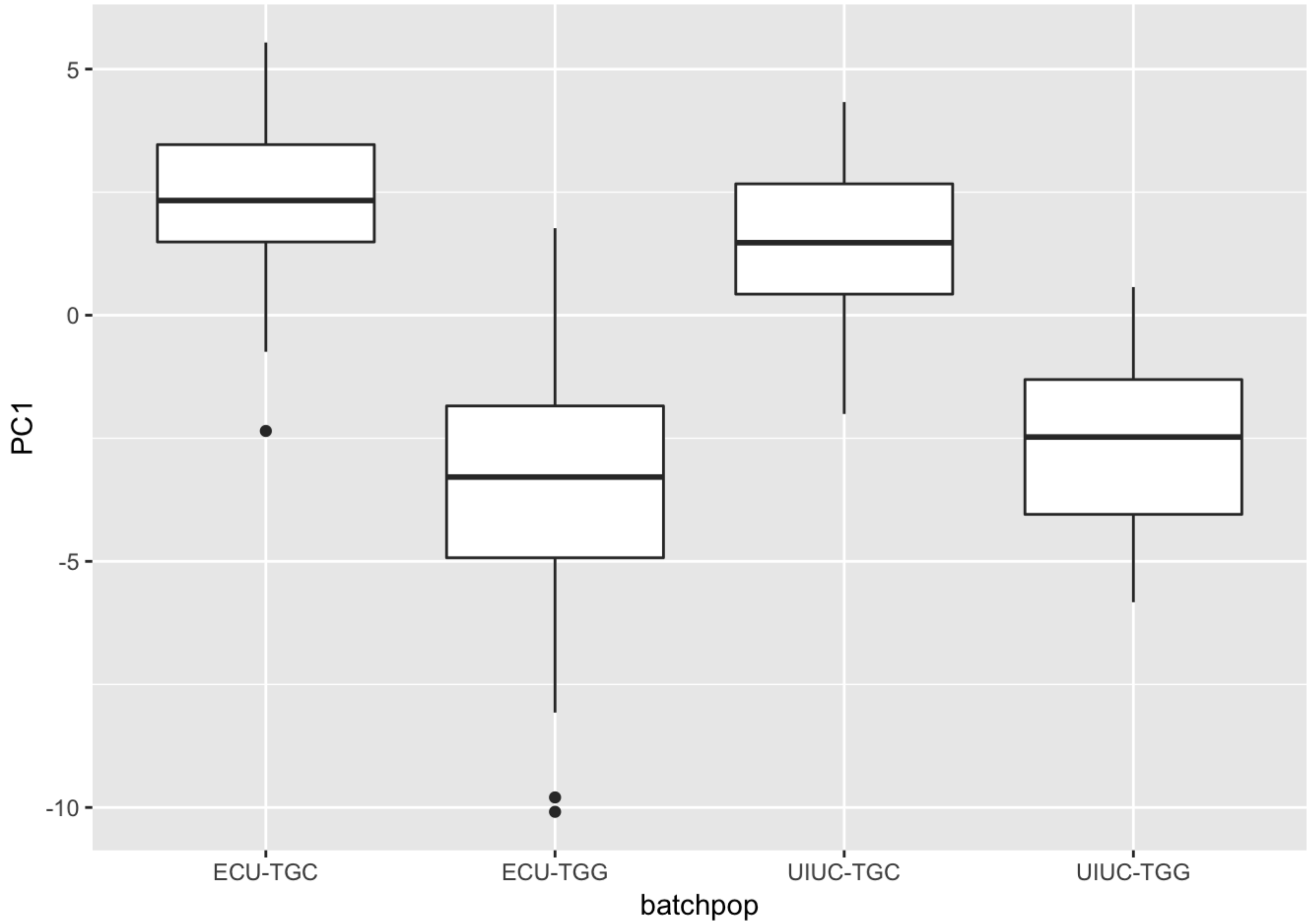

Superficially, we can clearly see that TGG is low variability in both populations. Lets test.

```
mPC1.TvA<- blmer(PC1 ~ batchpop + (1 | batchpop/Template) +  (1 | batchpop/Target), data = dat3, REML = FALSE)

VarCorr(mPC1.TvA) # Check for singularity
```

```
##  Groups            Name        Std.Dev.
##  Target.batchpop   (Intercept) 0.83666
##  Template.batchpop (Intercept) 1.50896
##  batchpop          (Intercept) 0.87592
##  batchpop.1        (Intercept) 0.87592
##  Residual                      1.17491
```

```
plot(mPC1.TvA) # residuals plot
```

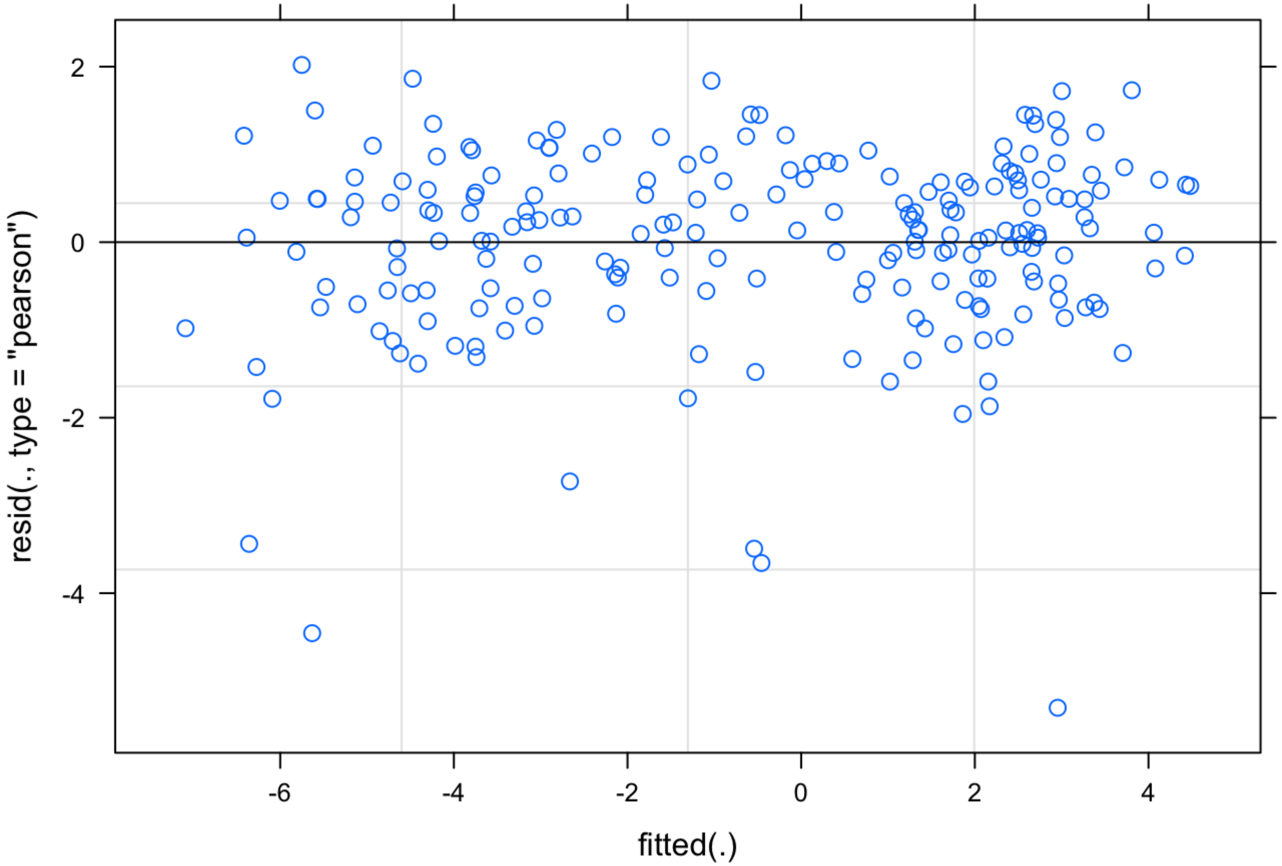


```
qqnorm(resid(mPC1.TvA)); qqline(resid(mPC1.TvA)) #qqplot
```

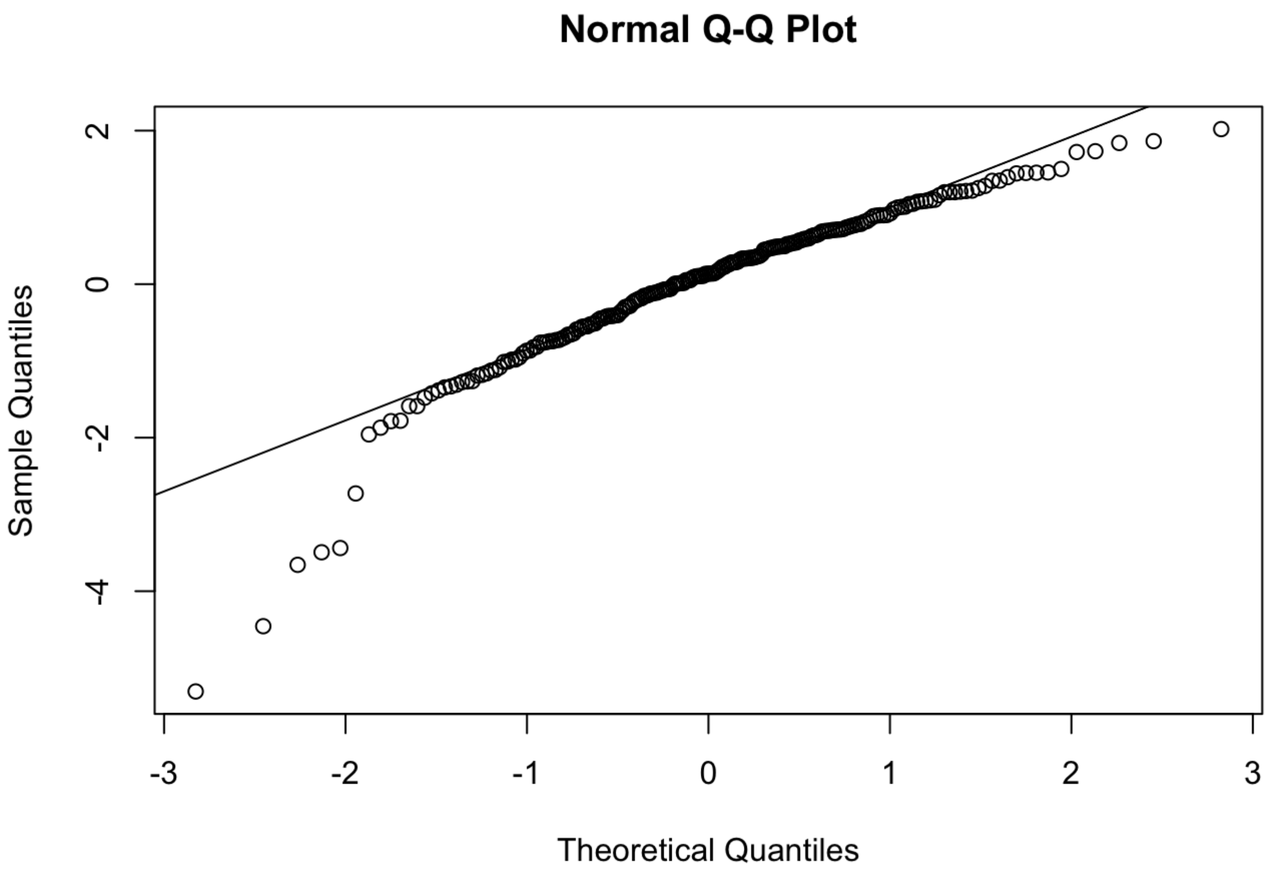


```
mPC1.TvA.null <- blmer(PC1 ~ 1 + (1 | batchpop/Template) + (1 | batchpop/Target), data = dat3, REML = FALSE)
VarCorr(mPC1.TvA.null) # Check for singularity
```

```
## Groups            Name        Std.Dev.
## Target.batchpop   (Intercept) 0.84285
## Template.batchpop (Intercept) 1.53347
## batchpop          (Intercept) 3.53784
## batchpop.1        (Intercept) 3.53784
## Residual                      1.17481
```

```
plot(mPC1.TvA.null) # residuals plot
```

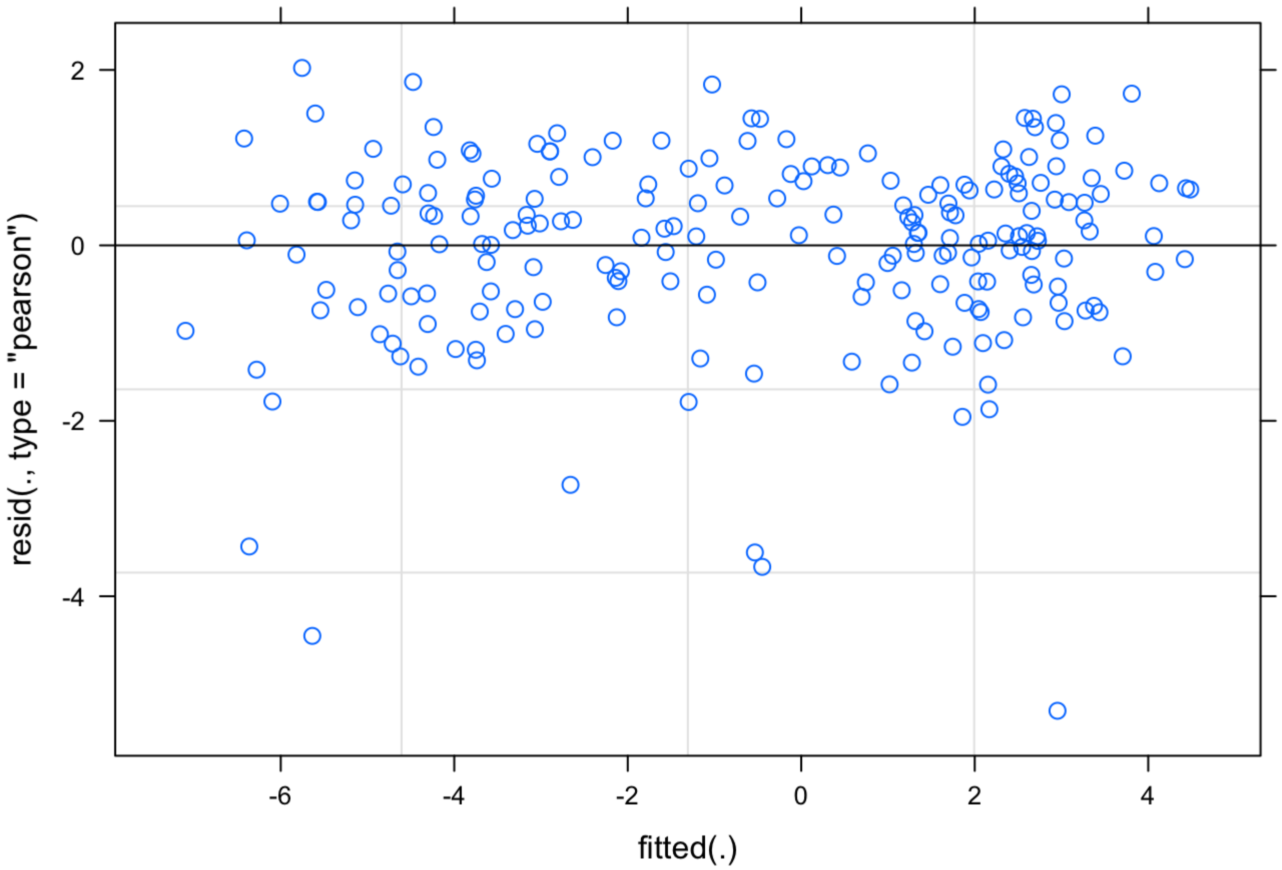


```
qqnorm(resid(mPC1.TvA.null)); qqline(resid(mPC1.TvA.null)) #qqplot
```

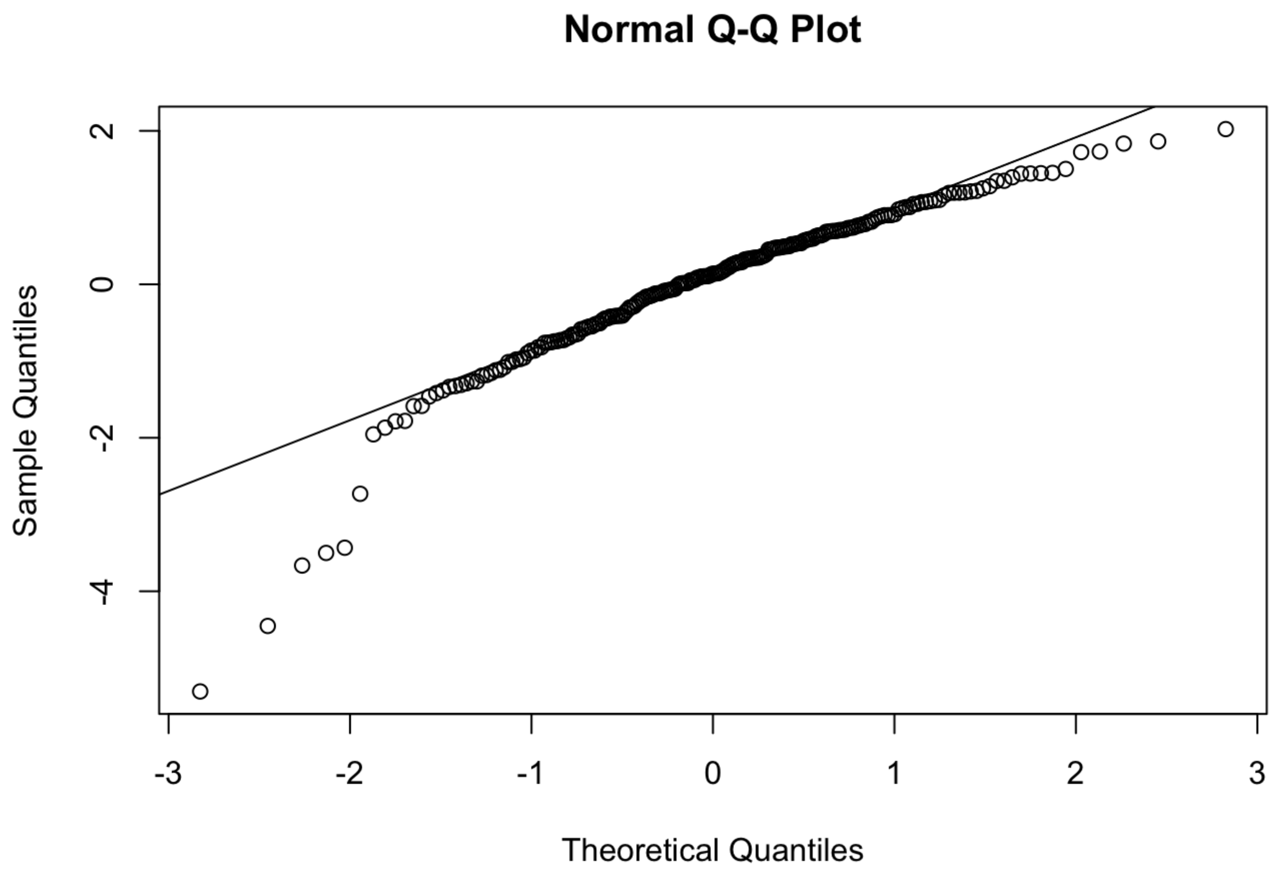


```
anova(mPC1.TvA,mPC1.TvA.null)
```

```
## Data: dat3
## Models:
## mPC1.TvA.null: PC1 ~ 1 + (1 | batchpop/Template) + (1 | batchpop/Target)
## mPC1.TvA: PC1 ~ batchpop + (1 | batchpop/Template) + (1 | batchpop/Target)
##               Df    AIC    BIC  logLik deviance  Chisq Chi Df Pr(>Chisq)
## mPC1.TvA.null  6 809.92 830.06 -398.96   797.92
## mPC1.TvA       9 804.85 835.06 -393.43   786.85 11.068      3    0.01136 *
## ---
## Signif. codes:  0 '***' 0.001 '**' 0.01 '*' 0.05 '.' 0.1 ' ' 1
```

```
dat3$predicted=(predict(mPC1.TvA,re.form=NA))
pframe <- data.frame(batchpop=dat3$batchpop)
dat3$LCI=as.vector((easyPredCInorm(mPC1.TvA,pframe))[,1])
dat3$UCI=as.vector((easyPredCInorm(mPC1.TvA,pframe))[,2])

ggplot(dat3,aes(x=batchpop,y=predicted))+geom_point(size=4)+geom_errorbar(aes(ymin=LCI,ymax=UCI),
                  width=0.4,
                  size=0.5)+
    ylab("PC1 (KL-Distance)")+xlab("Population")+theme_bw() +
  theme(axis.title=element_text(vjust=1,size=16,face="bold"), axis.text=element_text(size=14),
  axis.text.x = element_text(vjust=0.65, hjust=0.5, size=14), panel.border =
  element_rect(colour = "black",size=1.25)) + theme(axis.ticks.length=unit(0.3,"cm"))+
  theme(panel.grid.major = element_blank(), panel.grid.minor =element_blank(), axis.line =
    element_line(colour = "black"))
```

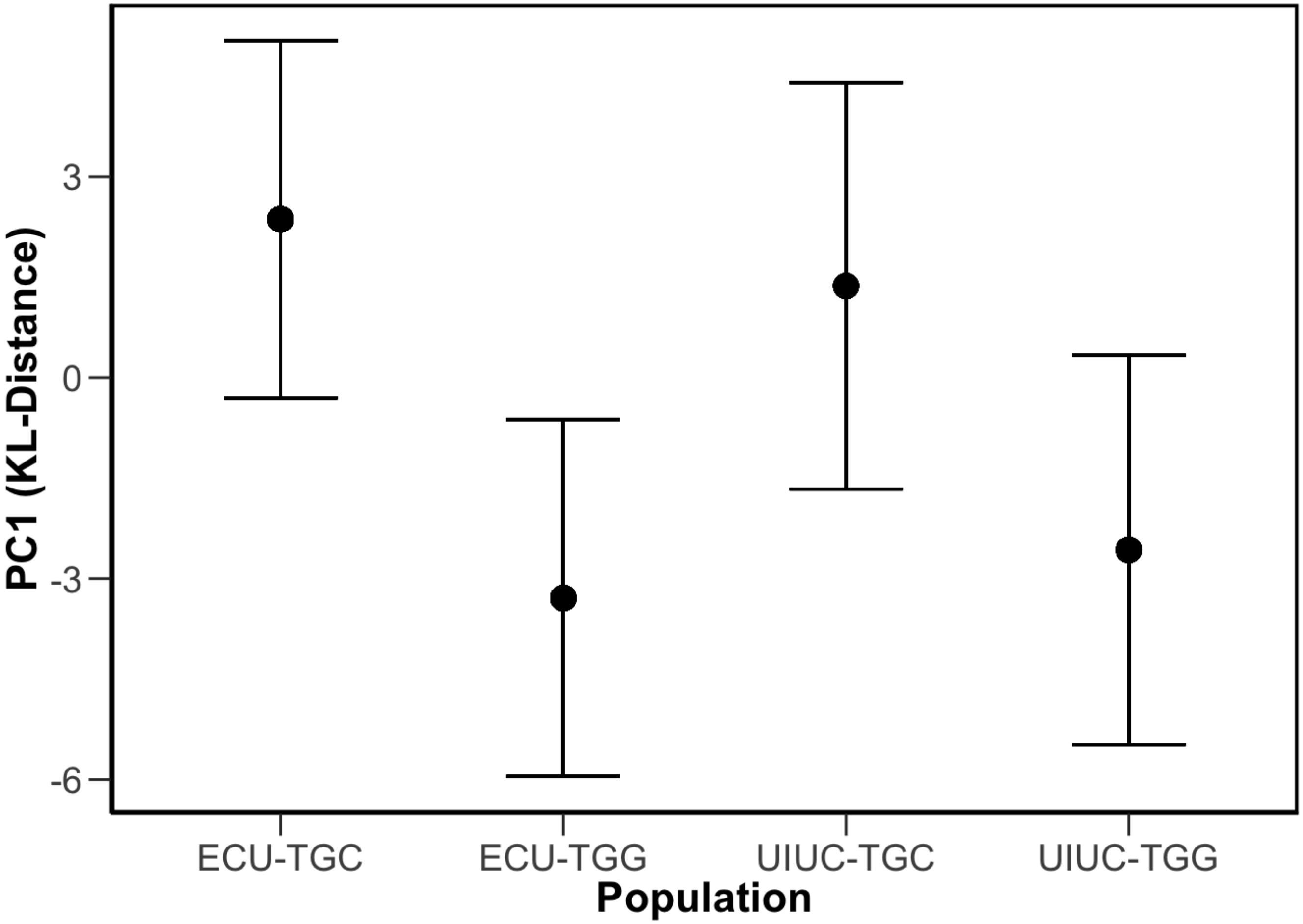


```
#ggsave("PC1-RplotTvA_4pop.pdf", plot=last_plot(), device=NULL, path=NULL, scale=1, width=NA, height=NA, dpi=300,
 limitsize=TRUE)
```

Now, the last model we wish to run deals with comparing TGC and TGG finches that were experimentally tutored by Bengalese finches. We first examine KL distances to tutors.

```
path<-"~/Documents/Projects/Domestication"
KLtoTutors=read.table(paste(path, "logKL_forPCAandMMA_Xfost.txt",sep="/"),sep = "\t",header=TRUE)
str(KLtoTutors)
```

```
## 'data.frame':    18 obs. of  27 variables:
##  $ logamplitude     : num  0.432 0.721 0.469 0.633 0.967 ...
##  $ logpitch         : num  0.399 0.517 0.274 0.322 0.923 ...
##  $ logFM            : num  0.55 0.279 0.524 0.337 0.585 ...
##  $ logAM2           : num  0.5575 0.0545 0.3093 0.0957 0.6293 ...
##  $ logentropy       : num  0.49 0.742 0.657 0.447 0.861 ...
##  $ logpitchgoodness : num  0.669 0.49 0.276 0.42 0.725 ...
##  $ logmeanfreq      : num  0.623 0.698 0.356 0.671 0.504 ...
##  $ logvpitch        : num  -0.0527 -0.3973 -0.2404 -0.5944 0.2876 ...
##  $ logvFM           : num  0.326 0.212 0.126 0.191 0.761 ...
##  $ logventropy      : num  0.1304 0.143 0.0728 0.0296 0.932 ...
##  $ logvpitchgoodness: num  0.232 0.232 -0.157 -0.103 0.368 ...
##  $ vmeanfreq        : num  0.0758 0.142 -0.0503 -0.0874 0.4318 ...
##  $ PC1              : num  1.268 0.663 -0.566 -0.894 4.553 ...
##  $ PC2              : num  -0.947 -1.255 -0.733 -1.513 0.714 ...
##  $ PC3              : num  0.79884 -1.21753 -0.00629 -0.32591 0.78802 ...
##  $ PC4              : num  0.466 0.269 0.671 0.406 0.196 ...
##  $ PC5              : num  -0.95 0.794 0.175 -0.363 1.261 ...
##  $ PC6              : num  0.22301 0.00671 0.2758 0.09311 0.23199 ...
##  $ PC7              : num  0.3141 0.5155 -0.9192 -0.0643 -0.2569 ...
##  $ PC8              : num  0.299 -0.189 0.879 -0.607 -0.558 ...
##  $ PC9              : num  -0.6274 0.0216 0.2184 0.2184 -0.1268 ...
##  $ PC10             : num  0.1677 0.1854 -0.2392 0.0494 -0.0106 ...
##  $ PC11             : num  0.2671 0.2916 0.2222 0.1099 -0.0342 ...
##  $ Population       : Factor w/ 2 levels "Aus","Timor": 1 1 1 1 1 1 1 1 1 1 ...
##  $ Stage            : Factor w/ 2 levels "egg","P30": 1 2 1 1 2 2 2 2 2 2 ...
##  $ Tutor            : Factor w/ 5 levels "B_BR90","B_DB10",..: 3 3 3 3 2 2 1 4 4 5 ...
##  $ Target.File      : Factor w/ 18 levels "filtered_BfADP56.xls",..: 3 6 9 10 4 8 5 1 2 7 ...
```

```
require(ggplot2)
print(ggplot(KLtoTutors,aes(x=Population,y=PC1))+geom_boxplot())
```

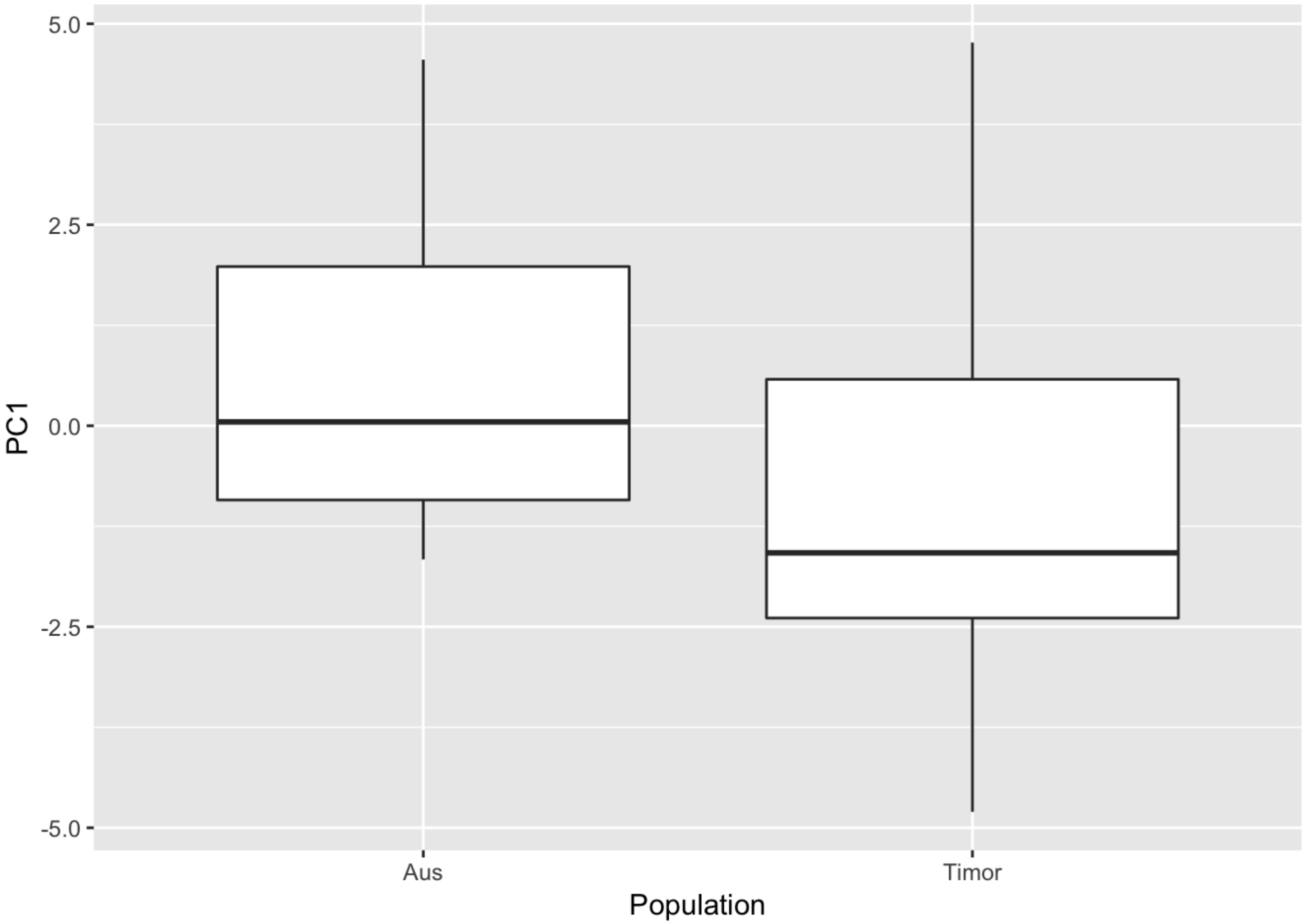

Ok, clearly not much exciting goin on here… but we should probably do stats anyway. Here Template is the Bengalese Finch Tutor

```
library(blme)
mKLtoTutor <- blmer(PC1 ~ Population + (1 | Tutor), data = KLtoTutors, REML = FALSE)
VarCorr(mKLtoTutor) # Check for singularity
```

```
##  Groups   Name        Std.Dev.
##  Tutor    (Intercept) 2.283
##  Residual             1.651
```

```
plot(mKLtoTutor) # residuals plot
```

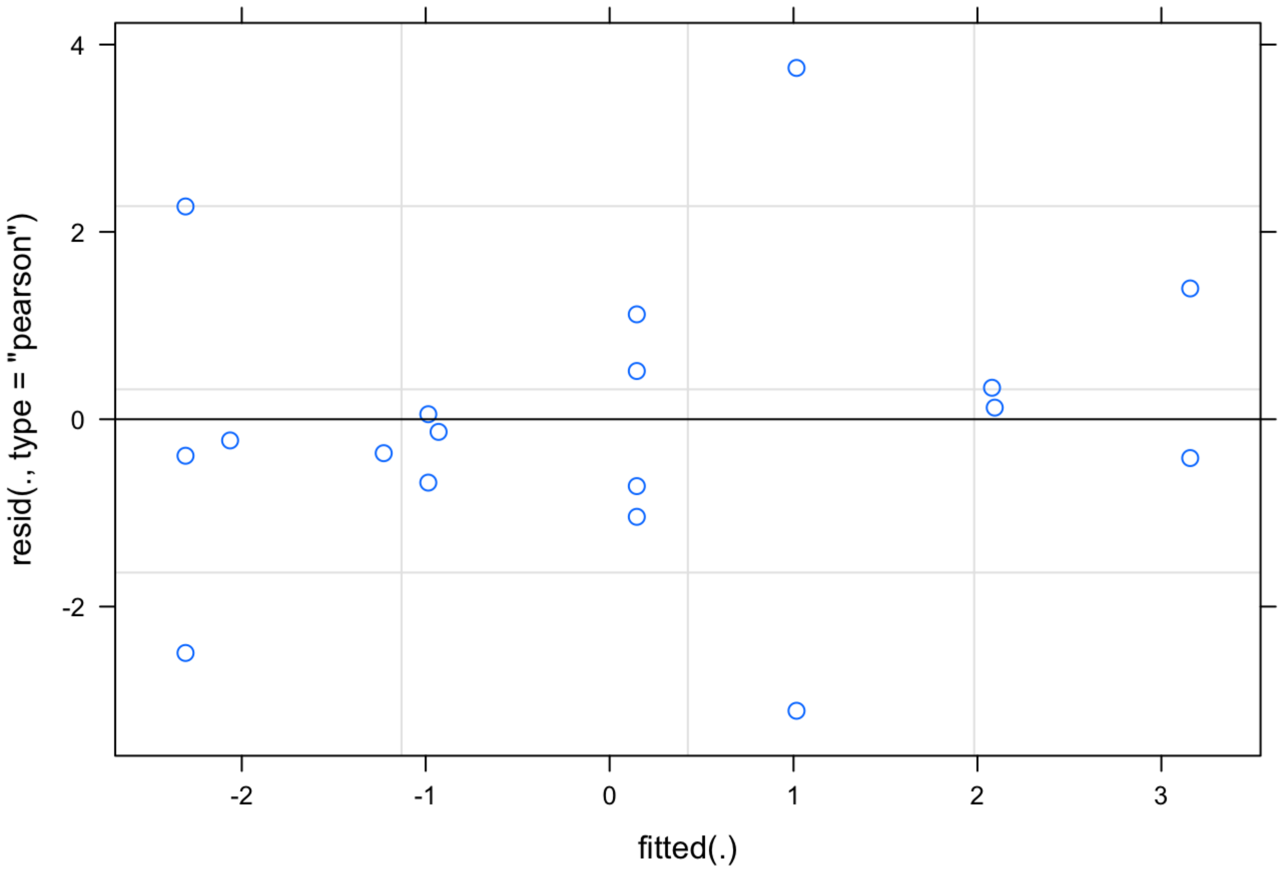


```
qqnorm(resid(mKLtoTutor)); qqline(resid(mKLtoTutor)) #qqplot
```

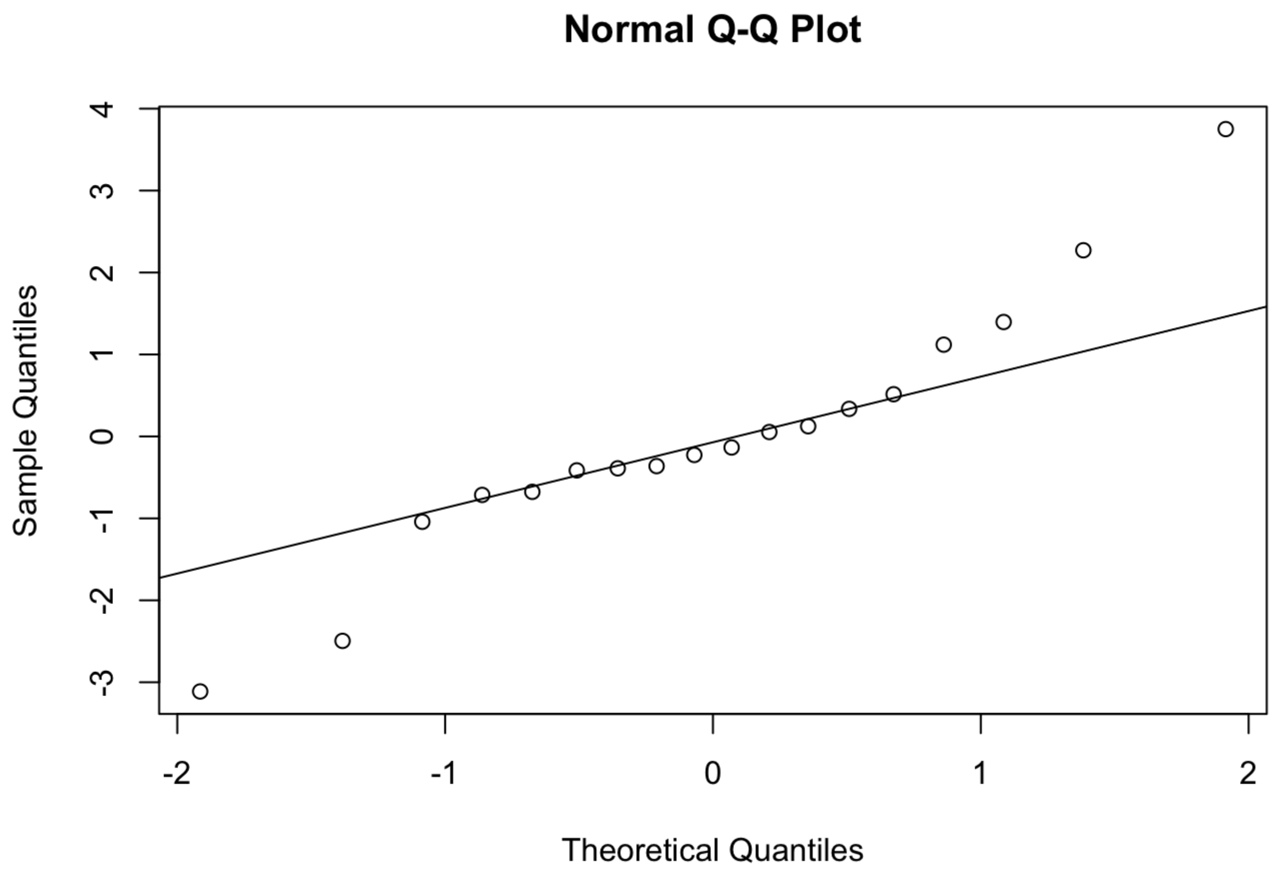


```
mKLtoTutorNull <- blmer(PC1 ~ 1 + (1 | Tutor), data = KLtoTutors, REML = FALSE)
VarCorr(mKLtoTutorNull) # Check for singularity
```

```
## Groups   Name        Std.Dev.
## Tutor    (Intercept) 2.3271
## Residual             1.7280
```

```
plot(mKLtoTutorNull) # residuals plot
```

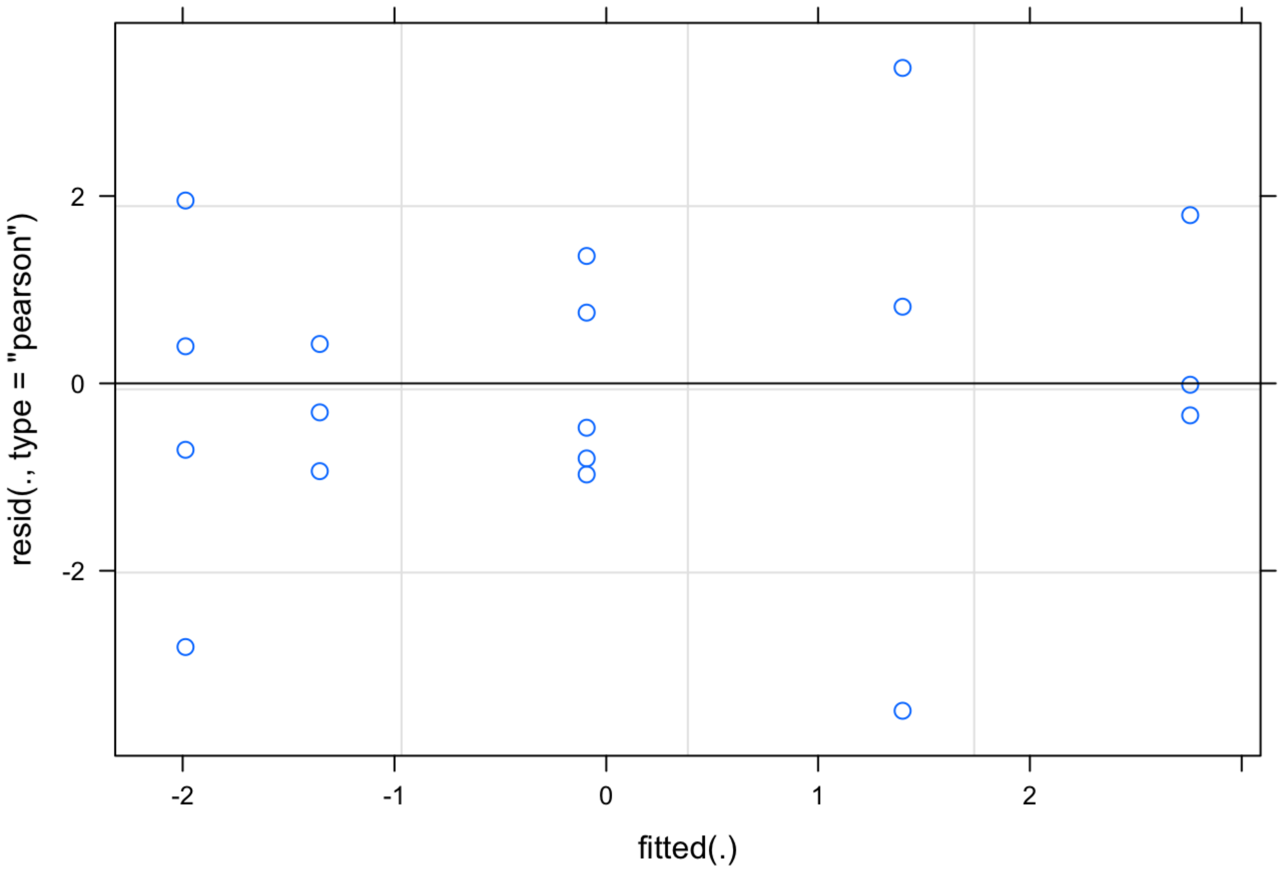


```
qqnorm(resid(mKLtoTutorNull)); qqline(resid(mKLtoTutorNull)) #qqplot
```

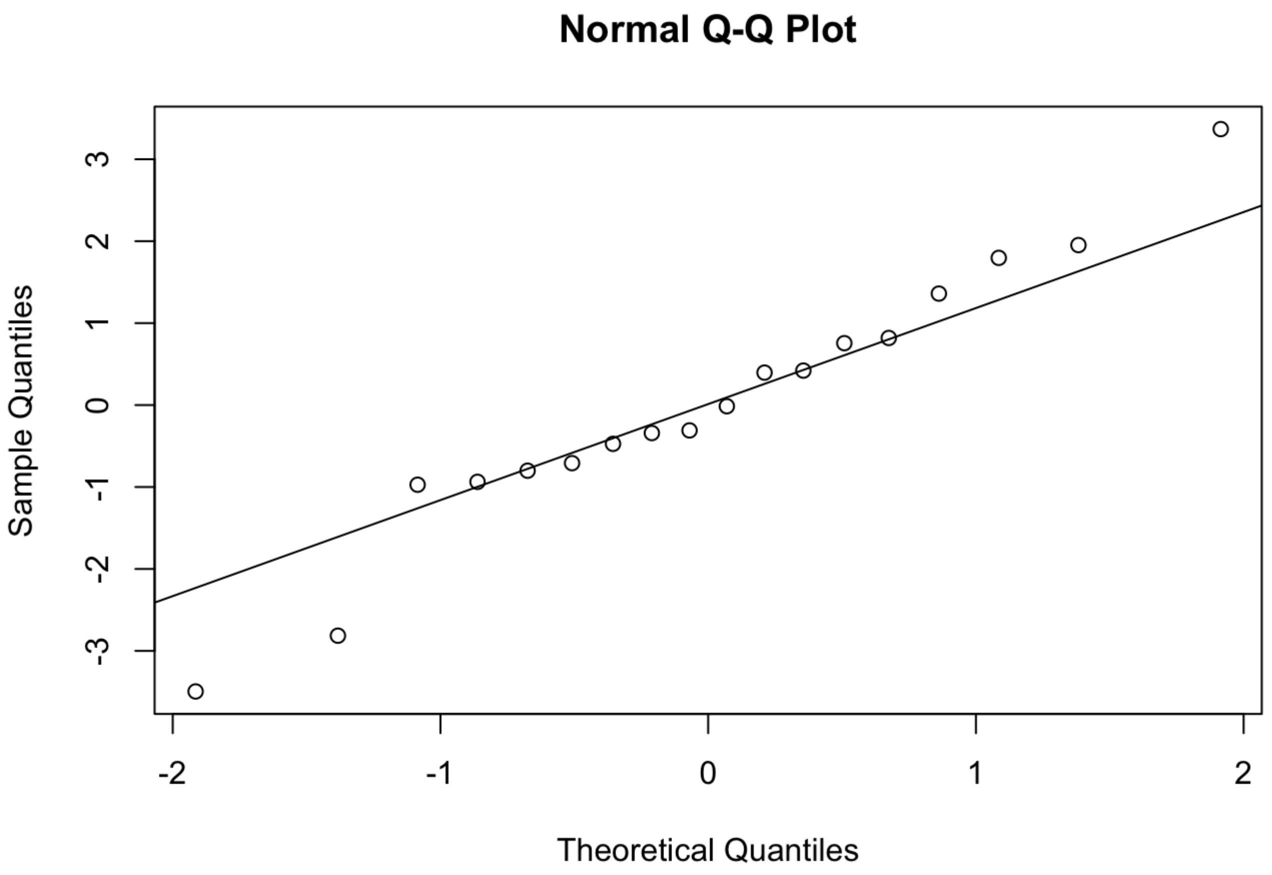

stats!

```
anova(mKLtoTutor,mKLtoTutorNull)
```

```
## Data: KLtoTutors
## Models:
## mKLtoTutorNull: PC1 ~ 1 + (1 | Tutor)
## mKLtoTutor: PC1 ~ Population + (1 | Tutor)
##                Df    AIC    BIC  logLik deviance Chisq Chi Df Pr(>Chisq)
## mKLtoTutorNull  3 86.780 89.451 -40.390   80.780
## mKLtoTutor      4 87.369 90.931 -39.685   79.369 1.411      1     0.2349
```

as predicted, no difference here. but lets plot anyway.

```
KLtoTutors$predicted=(predict(mKLtoTutor,re.form=NA))
pframe <- data.frame(Population=KLtoTutors$Population)
KLtoTutors$LCI=as.vector((easyPredCInorm(mKLtoTutor,pframe))[,1])
KLtoTutors$UCI=as.vector((easyPredCInorm(mKLtoTutor,pframe))[,2])

ggplot(KLtoTutors,aes(x=Population,y=predicted))+geom_point(size=4)+geom_errorbar(aes(ymin=LCI,ymax=UCI),
                  width=0.4,
                  size=0.5)+
    ylab("PC1 (KL-Distance)")+xlab("Population")+theme_bw() +
  theme(axis.title=element_text(vjust=1,size=16,face="bold"), axis.text=element_text(size=14),
  axis.text.x = element_text(vjust=0.65, hjust=0.5, size=14), panel.border =
  element_rect(colour = "black",size=1.25)) + theme(axis.ticks.length=unit(0.3,"cm"))+
  theme(panel.grid.major = element_blank(), panel.grid.minor =element_blank(), axis.line =
    element_line(colour = "black"))
```

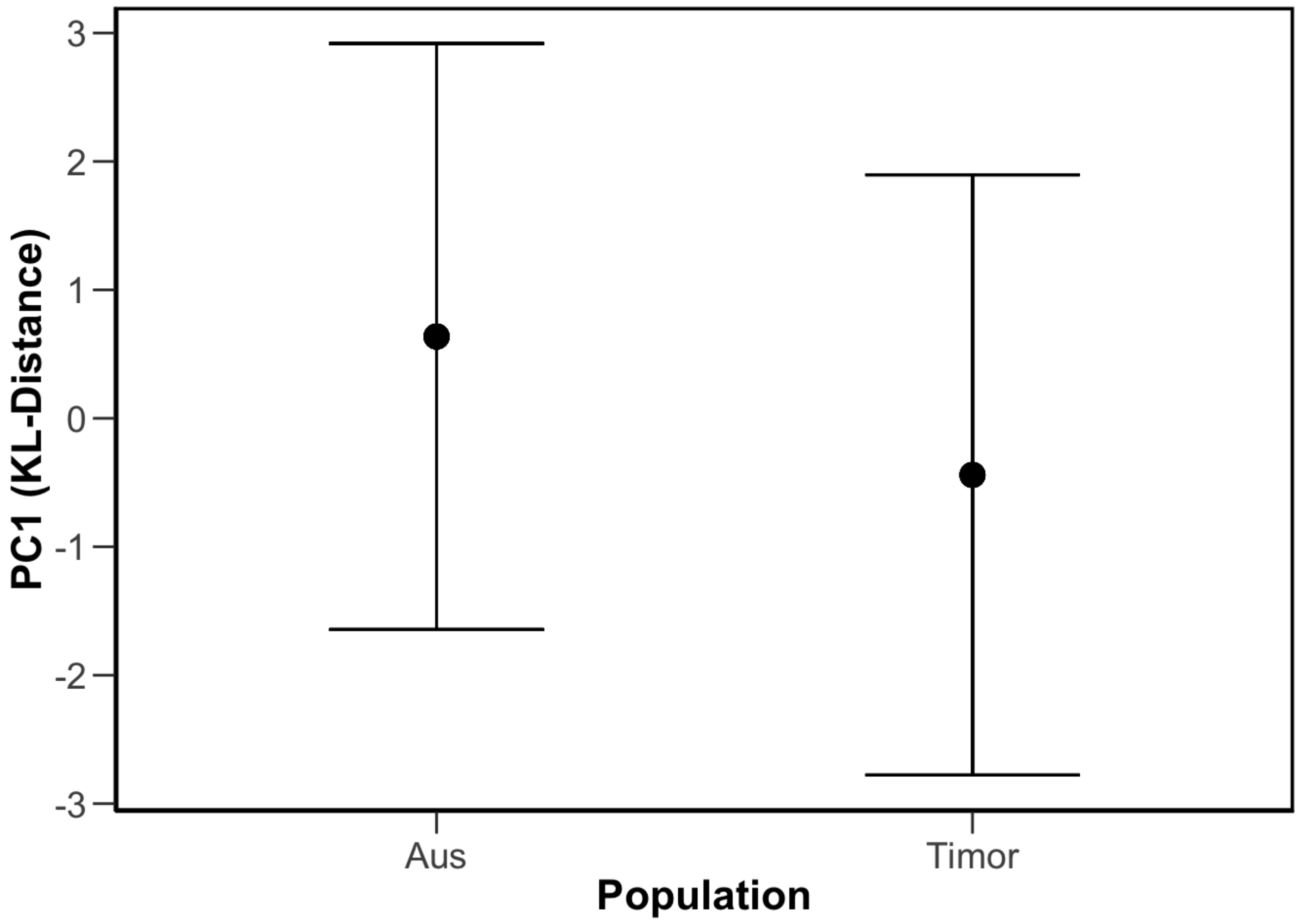


```
#ggsave("logPC1-KLxFosterToTutors.pdf", plot=last_plot(), device=NULL, path=NULL, scale=1, width=NA, height=NA, d
pi=300, limitsize=TRUE)
```

so the last thing we need to do is examine variability within the sets of birds that have been cross fostered.

```
path<-"~/Documents/Projects/Domestication/"
KLxfost=read.table(paste(path,"logXfostPairwiseWithinPops.txt",sep="/"),sep = "\t",header=TRUE)
str(KLxfost)
```

```
## 'data.frame':    20 obs. of  28 variables:
##  $ X                : int  1 2 3 4 5 6 7 8 9 10 ...
##  $ logamplitude     : num  -0.157 0.324 0.382 0.15 0.351 ...
##  $ logpitch         : num  0.0853 0.0279 0.0741 0.1064 0.1239 ...
##  $ logFM            : num  -0.15 0.32 0.117 0.108 0.565 ...
##  $ logAM2           : num  -0.115 -0.164 0.318 -0.289 0.2 ...
##  $ logentropy       : num  -0.2459 -0.0596 0.0853 -0.1641 0.0292 ...
##  $ logpitchgoodness : num  -0.0742 0.3476 0.0979 -0.0919 0.3875 ...
##  $ logmeanfreq      : num  -0.3064 -0.186 0.0188 -0.0168 0.2168 ...
##  $ logvpitch        : num  -1.287 -0.846 -0.281 -0.799 -0.639 ...
##  $ logvFM           : num  -0.5164 -0.2281 -0.1063 -0.2109 -0.0626 ...
##  $ logventropy      : num  -0.1112 -0.01312 -0.07199 -0.00938 0.03991 ...
##  $ logvpitchgoodness: num  -0.5112 -0.0907 -0.1372 -0.2589 -0.0846 ...
##  $ vmeanfreq        : num  -0.106 -0.392 -0.009 -0.274 0.315 ...
##  $ PC1              : num  -5.699 -3.149 -1.661 -4 -0.331 ...
##  $ PC2              : num  0.606 1.507 -1.114 0.483 0.456 ...
##  $ PC3              : num  -0.598 1.398 -0.178 0.706 -1.238 ...
##  $ PC4              : num  1.52 -1.284 -0.674 0.439 -1.433 ...
##  $ PC5              : num  0.703 -0.474 -0.259 -0.648 1.176 ...
##  $ PC6              : num  0.56 0.459 0.516 -0.567 -0.736 ...
##  $ PC7              : num  0.357 0.109 0.598 0.191 -0.282 ...
##  $ PC8              : num  0.4017 -0.2142 -0.0457 -0.4466 0.1006 ...
##  $ PC9              : num  0.0812 -0.0111 0.5832 -0.2039 0.054 ...
##  $ PC10             : num  0.0902 -0.1312 -0.0009 -0.317 -0.0206 ...
##  $ PC11             : num  -0.1156 -0.2461 0.2461 0.0657 -0.2288 ...
##  $ PC12             : num  -0.0774 0.1448 -0.1109 -0.1328 -0.0748 ...
##  $ Population       : Factor w/ 2 levels "ECU","Timor": 2 2 2 2 2 2 2 2 2 2 ...
##  $ Template         : Factor w/ 8 levels "filtered_BfAPU187.xls",..: 5 6 7 8 6 7 8 7 8 8 ...
##  $ Target           : Factor w/ 8 levels "filtered_BfADP56.xls",..: 5 5 5 5 6 6 6 7 7 8 ...
```

```r
require(ggplot2)
print(ggplot(KLxfost,aes(x=Population,y=PC1))+geom_boxplot())
```

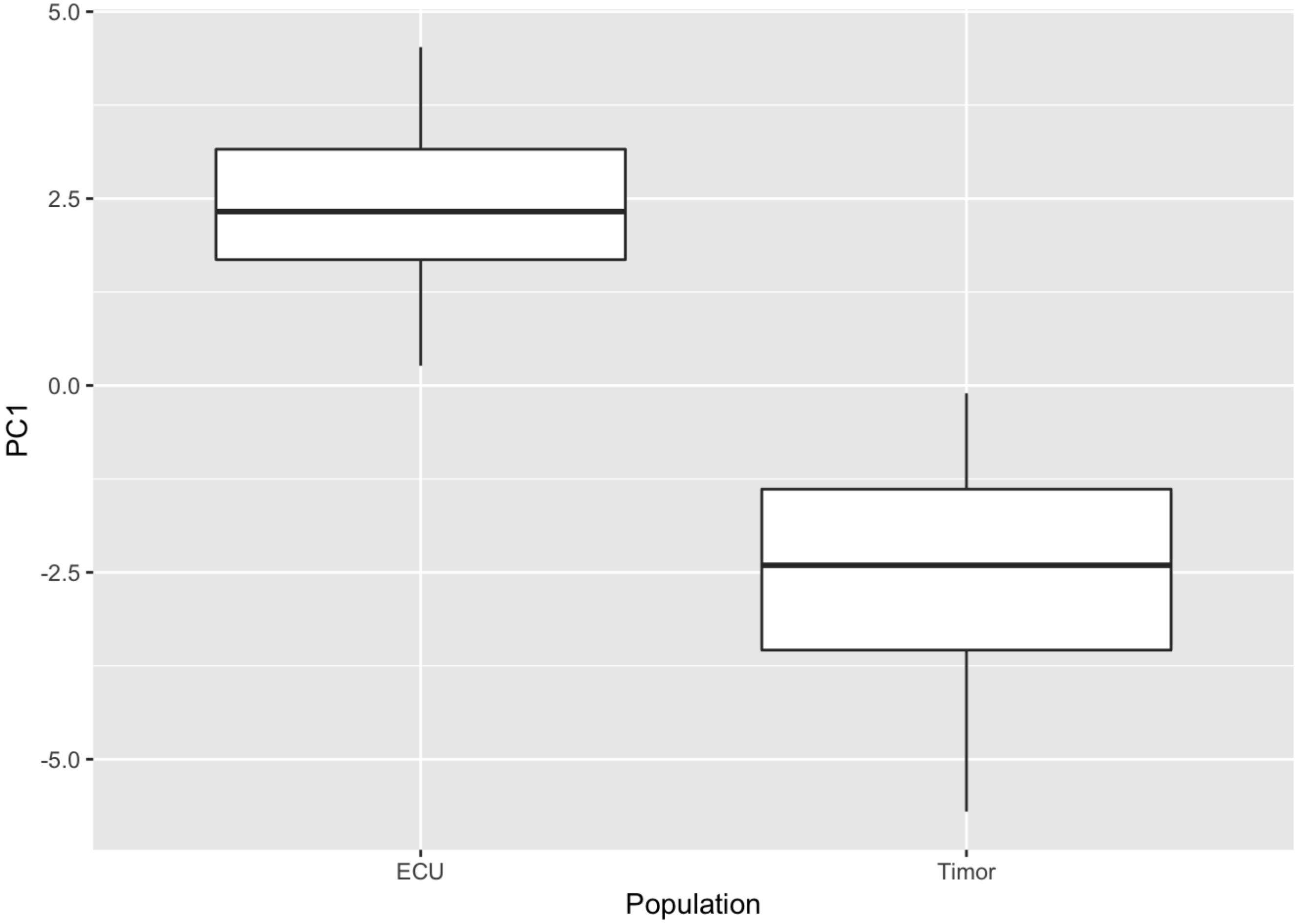

So here again we see a very similar pattern as weas described for the non cross fostered birds.

```
mPC1xFost <- blmer(PC1 ~ Population + (1 | Template) + (1 | Target) , data = KLxfost, REML = FALSE)
VarCorr(mPC1xFost) # Check for singularity
```

```
##  Groups   Name        Std.Dev.
##  Template (Intercept) 1.03509
##  Target   (Intercept) 1.07980
##  Residual             0.74016
```

```
plot(mPC1xFost) # residuals plot
```

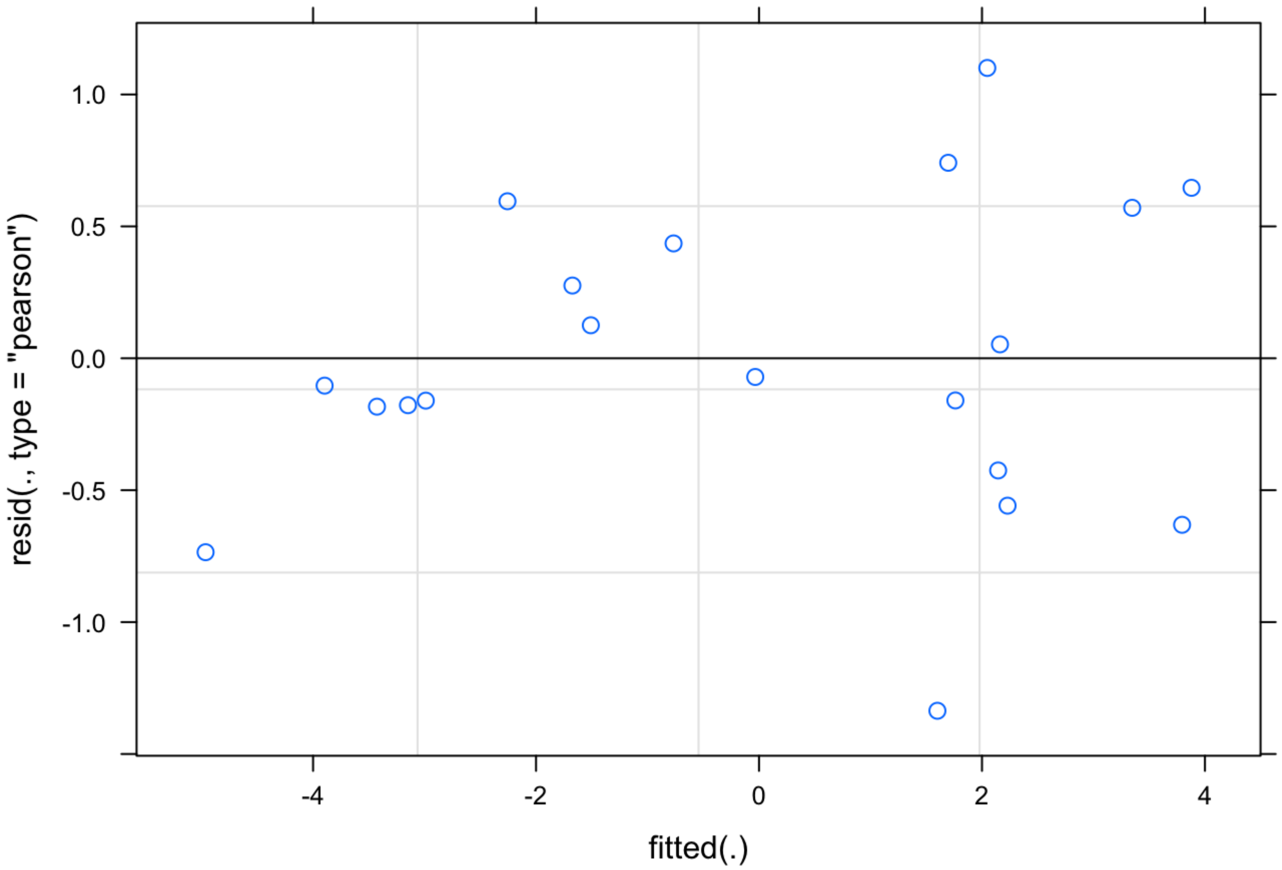


```
qqnorm(resid(mPC1xFost)); qqline(resid(mPC1xFost)) #qqplot
```

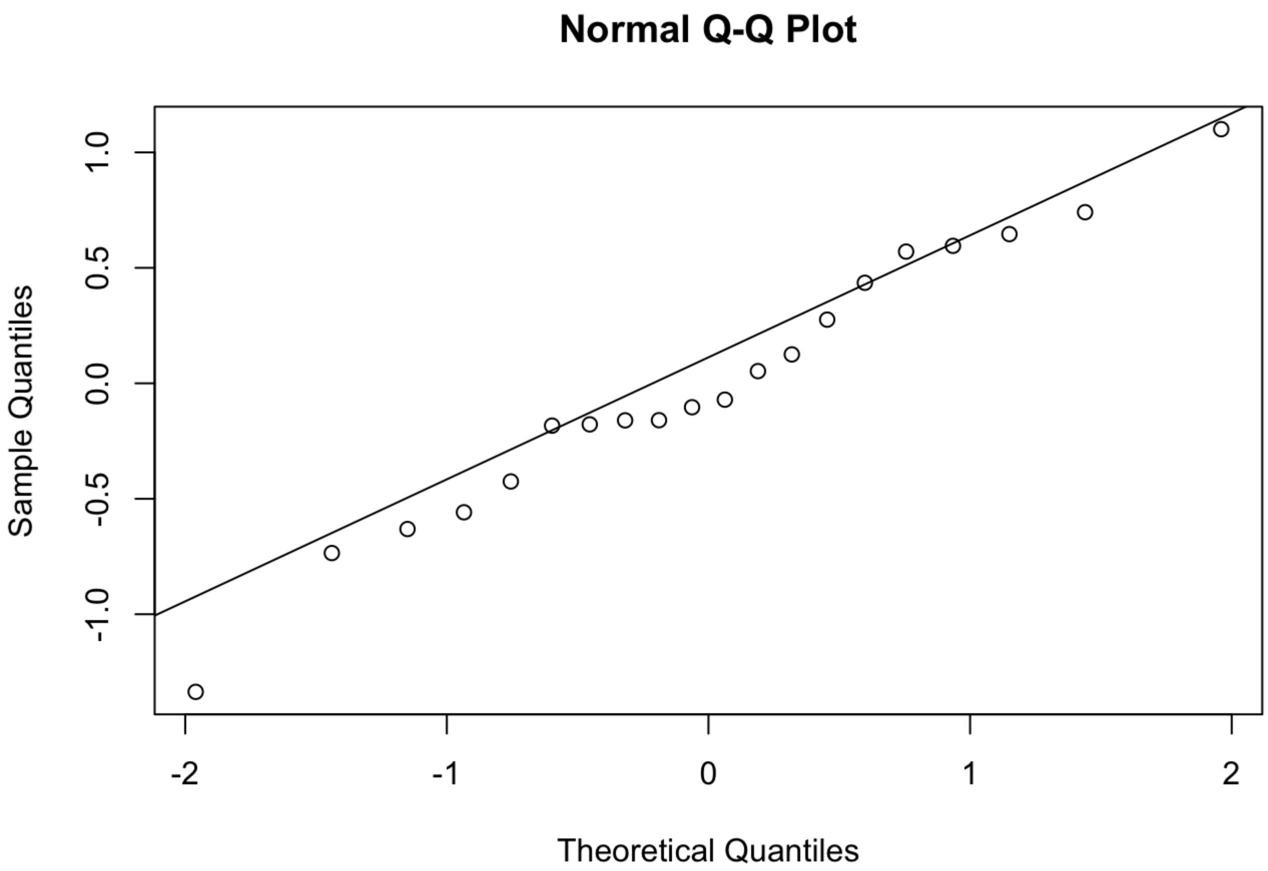


```
mPC1xFostNull <- blmer(PC1 ~ 1 + (1 | Template)+ (1 | Target), data = KLxfost, REML = FALSE)
VarCorr(mPC1xFostNull) # Check for singularity
```

```
##  Groups   Name        Std.Dev.
##  Template (Intercept) 1.2867
##  Target   (Intercept) 2.5255
##  Residual             0.7735
```

```
plot(mPC1xFostNull) # residuals plot
```

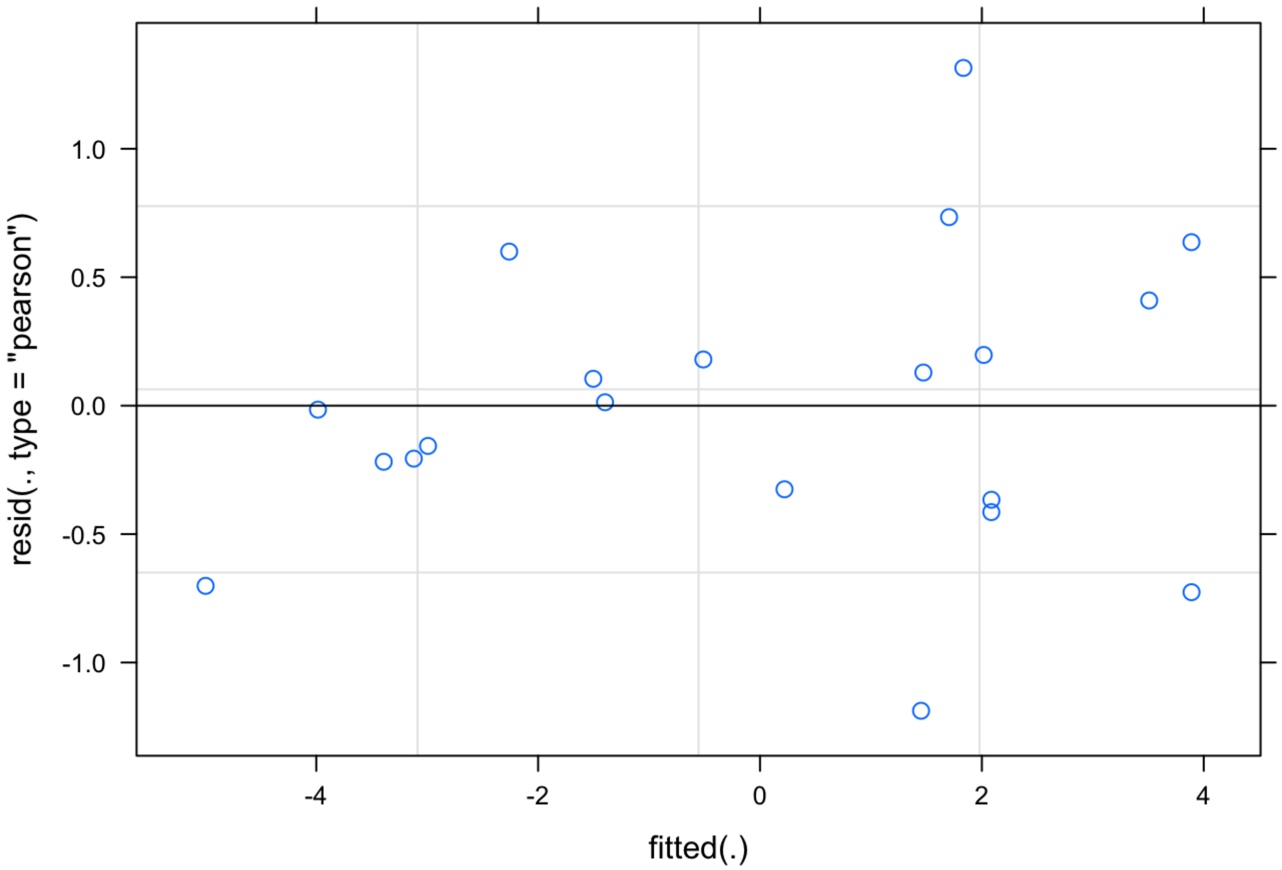


```
qqnorm(resid(mPC1xFostNull)); qqline(resid(mPC1xFostNull)) #qqplot
```

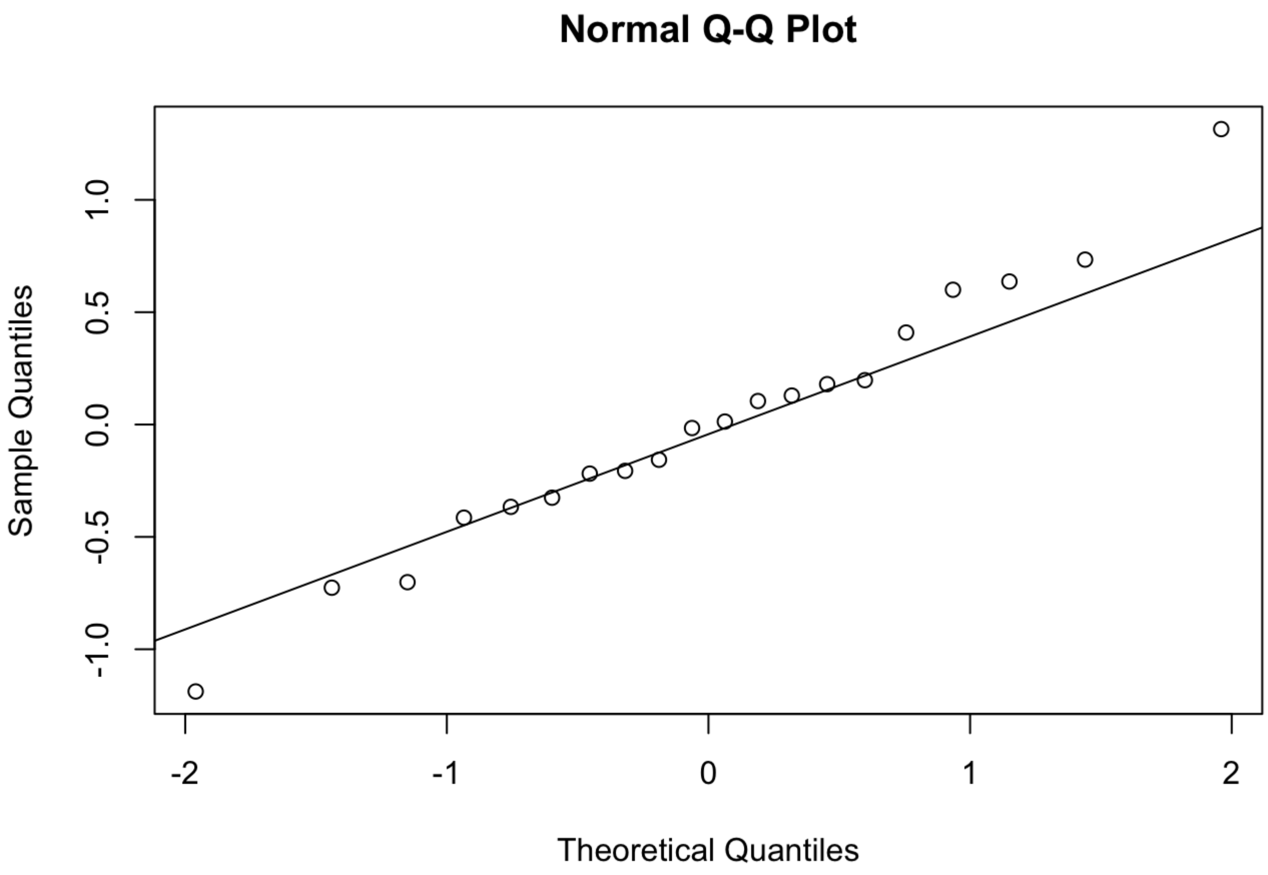

stats!

```
anova(mPC1xFostNull, mPC1xFost)
```

```
## Data: KLxfost
## Models:
## mPC1xFostNull: PC1 ~ 1 + (1 | Template) + (1 | Target)
## mPC1xFost: PC1 ~ Population + (1 | Template) + (1 | Target)
##               Df    AIC    BIC  logLik deviance  Chisq Chi Df Pr(>Chisq)
## mPC1xFostNull  4 91.535 95.518 -41.767   83.535
## mPC1xFost      5 79.379 84.358 -34.690   69.379 14.155      1  0.0001683
##
## mPC1xFostNull
## mPC1xFost     ***
## ---
## Signif. codes:  0 '***' 0.001 '**' 0.01 '*' 0.05 '.' 0.1 ' ' 1
```

```
KLxfost$predicted=(predict(mPC1xFost,re.form=NA))
pframe <- data.frame(Population=KLxfost$Population)
KLxfost$LCI=as.vector((easyPredCInorm(mPC1xFost,pframe))[,1])
KLxfost$UCI=as.vector((easyPredCInorm(mPC1xFost,pframe))[,2])

ggplot(KLxfost,aes(x=Population,y=predicted))+geom_point(size=4)+geom_errorbar(aes(ymin=LCI,ymax=UCI),
                 width=0.4,
                 size=0.5)+
    ylab("PC1 (KL-Distance)")+xlab("Population")+theme_bw() +
  theme(axis.title=element_text(vjust=1,size=16,face="bold"), axis.text=element_text(size=14),
  axis.text.x = element_text(vjust=0.65, hjust=0.5, size=14), panel.border =
  element_rect(colour = "black",size=1.25)) + theme(axis.ticks.length=unit(0.3,"cm"))+
  theme(panel.grid.major = element_blank(), panel.grid.minor =element_blank(), axis.line =
    element_line(colour = "black"))
```

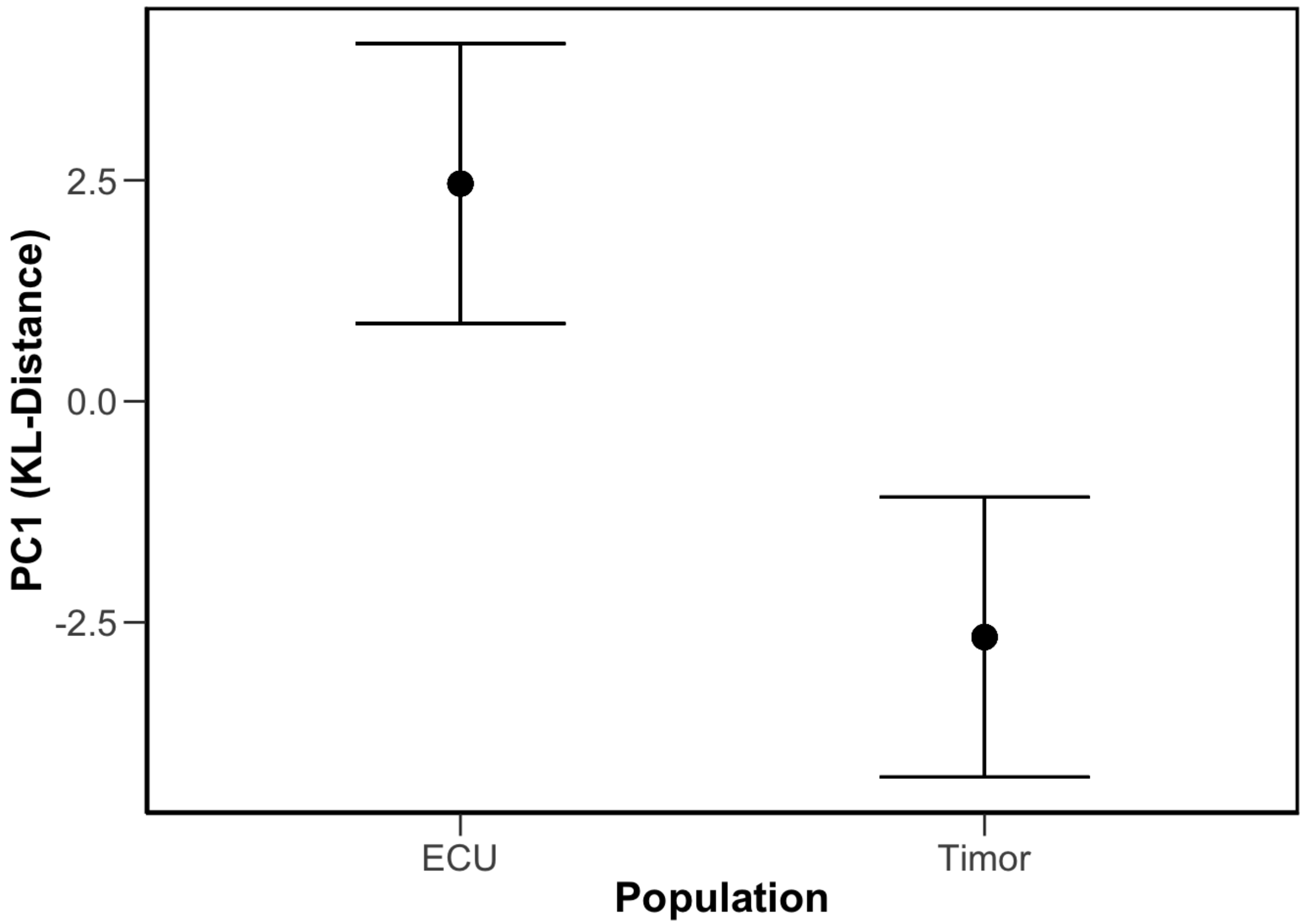


```
#ggsave("PC1-KLxFosterWithin.pdf", plot=last_plot(), device=NULL, path=NULL, scale=1, width=NA, height=NA, dpi=30
0, limitsize=TRUE)
```
